# Pension Wealth and the Gender Wealth Gap

**DOI:** 10.1007/s10680-022-09631-6

**Published:** 2022-08-22

**Authors:** Karla Cordova, Markus M. Grabka, Eva Sierminska

**Affiliations:** 1 Pomona College, Claremont, USA; 2 DIW Berlin, Berlin, Germany; 3 Luxembourg Institute of Socio-Economic Research (LISER), DIW, IZA and GLO, Esch/Alzette, Luxembourg

**Keywords:** Gender wealth gap, Pension entitlements, Germany, Redistribution, SOEP Socio-Economic Panel (SOEP), H55, D31, J16

## Abstract

We examine the gender wealth gap with a focus on pension wealth and statutory pension rights. By taking into account employment characteristics of women and men, we are able to identify the extent to which the redistributive effect of pension rights reduces the gender wealth gap. The data for our analysis come from the German Socio-Economic Panel (SOEP), one of the few surveys that collects information on wealth and pension entitlements at the individual level. Pension wealth data are available in the SOEP for 2012 only. While the relative raw gender wealth gap is about 35% (or 31,000 euros) when analysing the standard measure of net worth, it shrinks to 28% when pension wealth is added. This reduction is due to redistributive elements such as caregiver credits provided through the statutory pension scheme. Results of a recentred influence functions (RIF) decomposition show that pension wealth reduces the gap substantially in the lower half of the distribution. At the 90th percentile, the gender wealth gap in net worth and in augmented wealth remains more stable at roughly 27–30%.

## Introduction

Employment plays an important role in determining private wealth accumulation. Not only does it provide income that can be saved to build wealth, but it also enables the accrual of pension rights, as the majority of pension systems are earnings-related (Frey, 2021, p. 123). Given that women still earn less than men on average, it comes as no surprise that the gender wealth gap widens when taking pension assets (the present value of all pension entitlements from statutory and occupational pension schemes) into account. The average gap in net wealth between working-age men and women in Germany was 31,000 euros in 2012 and widens to around 45,000 euros when pension assets are added.^1^

In this paper, we examine the gender wealth gap with a focus on pension wealth and statutory pension rights. By taking into account the employment histories of women and men, we are able to measure the extent to which gender-specific disadvantages of women in the labour market—for instance, the pay gap (Blau & Kahn, 2017), the glass ceiling effect (Biagetti & Scicchitano, 2011), and the motherhood penalty (Anderson et al., 2002)—reduce the accumulation of net worth. Moreover, we can determine whether these negative effects are further reinforced when pension wealth is considered. Since the old-age pension system in Germany is based strictly on the equivalence principle, pension entitlements *de facto* directly reflect women’s employment and career trajectories and thus their disadvantageous situation in the labour market. This situation is countered by redistributive elements of the statutory pension system that are intended to compensate women for employment interruptions, for instance, by granting caregiver credits for periods of child-rearing (Bonnet & Rapoport, 2020). The question arises to what extent these redistributive elements are able to compensate for the disadvantages women experience.

The case of Germany is also of interest from another perspective. Since the end of the Second World War up to German reunification in 1989, East and West Germany differed significantly in their development. Even today, there are relevant cultural, normative, and economic differences between the two formerly separate parts of Germany. When it comes to gender differences, the male-breadwinner model was and in some cases still is predominant in the former West, whereas a dual-earner model predominates in the former East (Trappe et al., 2015). One important continuing difference is in women’s labour market participation. In 1989, women in the German Democratic Republic (GDR) had one of the highest rates of labour market participation in the world, at 91.3%, compared to just 51% in the Federal Republic of Germany (FRG) (Wippermann, 2015).^2^ These general structural differences persist and are expressed in the wage gap: In 2018, this gap was 22% in the former West compared to 7% in the former East (Destatis, 2020). Labour market differences are also reflected in the opportunities conducive to accumulating pension assets. Occupational pensions are overall much less prevalent in the East, yet in 2018, the gender pension gap among retired people was 55% in the West and 23% in the East (BMAS, 2020). The empirical data for the present study come from the Socio-Economic Panel (SOEP), one of the few datasets containing information on wealth as well as pension entitlements at the individual level. Pension wealth data is available in the SOEP for 2012 only. Individual-level wealth data allow us to analyse the gender wealth gap between women and men across all households. Thanks to the longitudinal character of the SOEP data, we are also able to consider detailed information on employment trajectories and family-related events (such as childbirth, marriage, divorce, and widowhood) that can have an effect on (public) pension entitlements. We assume a simple model of wealth accumulation in which assets in each period are the result of the stock of assets in the previous period augmented by savings (income minus consumption) and the gross rate of return (depicted in (Davies & Shorrocks, 2000), (Sierminska et al., 2010)). Individuals differ in wealth accumulation for several reasons. First, different individuals start off with different stocks of assets, in some cases due to intergenerational transfers (inheritances and bequests), and they also differ in their ability and willingness to save. Such differences are often substantial between women and men and are discussed further in Sect. 5. Previous research focused primarily on the standard measure of net worth when analysing the gender wealth gap. This paper is—as far as we know—the first to consider pension wealth to analyse this gap. Pension wealth consists of three components: statutory public pensions, occupational pensions, and private pensions. Although private pensions are often taken into account in the standard measure of net worth, there is often no survey data available on statutory public pension entitlements or occupational pensions, leading to their neglect in the analysis of the gender wealth gap. The distinction between private pensions on the one hand and statutory public and occupational pensions on the other is necessary for several reasons. Whereas private pensions are based solely on a voluntary investment decision by an individual, statutory pension insurance is tied to employment and is compulsory for employees who are subject to social security contributions. Occupational pensions are also tied to employment, but they are not always provided by employers. Furthermore, both statutory and occupational pensions cannot be sold or used as collateral, meaning that the usual functions of wealth, except for the security function, are not fulfilled. Despite this, as Bönke et al. (2019) show in the case of Germany, employees accumulate comparable amounts of statutory public and occupational pensions to what they accumulate in net worth. This underscores the importance of considering the former two pension types when analysing differences in wealth between groups. In the empirical part of this paper, we characterise gender differences in net wealth and then present an augmented measure of private wealth that includes pension wealth. We then decompose the gender wealth gap using the Oaxaca–Blinder decomposition (Oaxaca, 1973; Blinder, 1973) at the mean and throughout parts of the distribution following (Firpo et al., 2009). Then, we examine the distribution of pension wealth by type and test the robustness of our results, conducting the estimations for several subsamples. The structure of the paper is as follows. Section 2 reviews the literature on the gender pension wealth gap, and Sect. 3 describes the characteristics of the German pension system. Section 4 discusses the data and is followed by Sect. 5 on the analytical framework and empirical strategy. In Sect. 6, we provide a descriptive analysis of the wealth data and individual characteristics. The results for the mean and detailed decomposition are found in Sect. 7 for each of the wealth aggregates, as well as for different pension types and sub-samples. Finally, we conclude in Sect. 9 and discuss possible policy implications and future steps.

## Literature Review

Research on gender differences in private or pension wealth is usually confronted with a lack of individual-level wealth data, which has meant that only a few papers to date have been able to analyse the gender gap in private wealth or pension wealth. One of the few representative population surveys to collect wealth information at the individual level is the German SOEP. Several papers make use of SOEP data to describe gender differences in wealth levels or wealth changes, including Frick et al. (2007), Sierminska et al. (2010), Grabka et al. (2015), Lersch (2017a, b), Boertien and Lersch (2021), and Kapelle and Baxter (2021). These authors show that there is a significant gender gap in private wealth in Germany, not only between single men and women, but even within married couples. The main driver of the gender gap in private wealth are differences in labour market outcomes such as participation in the labour market and earnings levels. Individuals who work in stable, full-time, higher-prestige occupations will consistently earn more (and have higher permanent income), which will improve their ability to save (Ruel & Hauser, 2013). Women’s lower labour market participation rate, their lower working hours, the glass ceiling effect, and the still existing gender pay gap all hinder women’s wealth accumulation (Warren et al., 2001). In addition, there is vertical and horizontal segregation between men and women in the labour market that contributes to the gender pay gap^3^ and consequently to the wealth gap. Women face a motherhood penalty in wages (Anderson et al., 2002) due to gender stereotypes and assumptions about traditional roles in the family (e.g. (Lewis, 1992)). However, women’s labour force participation is also significantly influenced by the availability and quality of childcare facilities (Kreyenfeld & Hank, 2000). Finally, the fiscal regime matters. In many OECD countries, couples’ incomes are pooled for tax purposes, which implies that the tax rate on second earners remains significantly higher than on single individuals, which has a negative impact on labour force participation of the lower earner (Jaumotte, 2004). Besides labour market differences, intergenerational transfers play an important role in wealth accumulation. However, several papers show that there are no systematic gender differences in the amount of inheritance received (e.g. (Ruel & Hauser, 2013)). Women and men also show different levels of returns from their investments due to diverging risk preferences, which results in different wealth portfolios (Sunden & Surette, 1998; Chang, 2010; Lersch, 2017b). For example, women are significantly less likely to own business assets (e.g. Austen et al. 2014) and are more likely to own property. As Goldsmith-Pinkham and Shue (2020) argue, the gender gap in housing returns can explain 30% of the gender gap in wealth accumulation at retirement. Access to credit (Alesina et al., 2013) and mortgages (e.g. Goldsmith-Pinkham and Shue 2020) may differ between women and men, which affects their ability to accumulate additional wealth. Additionally, financial literacy influences investment decisions (Huston, 2010; Lusardi & Mitchell, 2008), and it has been shown that women have lower financial knowledge than men, which leads them to have more conservative investment patterns and thus lower (that is, safer) returns than men (Almenberg & Dreber, 2015). Another aspect that could affect wealth levels are marital status transitions. The dissolution of marriage is negatively related to the accumulation of wealth over time, and the side effects are similar for both genders. However, the dissolution of cohabiting unions is accompanied by wealth losses for women but not for men (Boertien & Lersch, 2021). In addition, parenthood, within or outside of marriage, has a negative effect on women’s employment and wages and thus impairs their individual wealth accumulation (Yamokoski & Keister, 2006; Lersch, 2017b).^4^ The presentation so far relates to drivers of gender differences in private wealth. When it comes to gender differences in pension wealth, similar but also additional aspects come to light. First, a large number of papers investigate membership in occupational or private pension plans. Rõõm and Soosaar (2021) show that in the euro area, more men than women have pension wealth from defined contribution (DC) pension plans. The raw gap in the value of pension wealth is 65% of the mean value of women’s pension wealth, which is considerably larger than the average gender wage gap in Europe. When the authors control for observable characteristics, this gap shrinks to 9%.

Gender differences in private pension wealth are more pronounced. Not only do women contribute less to private pension schemes (e.g. (Foster & Smetherham, 2013), (Gardiner et al., 2016)), the gender gap in mean values is also significantly larger for private pensions. Johnson et al. (1999), for example, find that full-time workers’ median pension wealth for their current job is 76% greater for men than for women. Differences in age, occupational position, earning levels, working hours, and having dependent children in the household account for most of the gender gap in pension wealth. For statutory pensions, the picture is quite different. Although public pension systems are often earnings-related, which means that the gender pay gap translates into a gender pension gap, there are several redistributive elements in favour of women that dampen the effect. In the case of Switzerland, Kuhn (2020) notes that women tend to have higher pension wealth from statutory pensions, which is due to a weak relationship between earnings and statutory pension levels there. In other countries, the contribution ceiling favours women, as fewer women earn above the threshold. In addition, statutory pension schemes usually have strong redistributive elements to benefit women. This is true in particular for caregiver credits. In the Norwegian pension scheme, for instance, these have the strongest effect on reducing the gender pension wealth gap (Halvorsen & Pedersen, 2019). In France, caregiver credits almost completely offset the differences in pension entitlements between mothers and non-mothers, but not those between genders (Bonnet & Rapoport, 2020). However, in the majority of European countries, caregiver credits are not able to compensate for the motherhood penalty that is accrued over the course of working life (Möhring, 2018). Looking at the differences between East and West Germany, net wealth is significantly higher in the West than in the East (Grabka, 2014). This is due, on the one hand, to historical conditions: In the East prior to German reunification, there was no opportunity to invest in companies or shares and little opportunity to buy real estate, which hampered wealth accumulation. On the other hand, this difference is due to demographic developments. Large parts of East Germany still face population decline, which has a negative effect on real estate prices. With respect to pension wealth, the picture is more mixed. While for statutory pensions, male (female) pensioners in East Germany receive about 6% (44%) higher gross pensions than their peers in West Germany, the respective figure for occupational pensions is −54% (−45%) (BMAS, 2020). Not only are occupational pensions significantly lower in East Germany, they are also much less prevalent. Although women in the East have a higher rate of labour force participation and work longer hours on average than women in the West, wage levels in the East are still lower. Thus, ultimately, what happens to the gender wealth gap in these two regions when pension entitlements are taken into account is an empirical question.

## The German Pension System

The German pension system consists of three pillars. The first pillar is the statutory public pension scheme, consisting of statutory pension insurance, civil servant, and liberal profession pension insurance. The second pillar is the occupational pension scheme. In these two pillars, insured individuals acquire pension entitlements throughout their working careers. Following the principle of equivalence, pension entitlements from the first and second pillars are proportionate to overall life-cycle earnings during the active phase of working life. The third pillar consists of private voluntary insurance plans (for an overview of old-age security policy in Germany, see (Schmähl, 2018)).

*First Pillar: The Statutory Public Pension Scheme*

*Statutory pension insurance* About three-quarters of the German working-age population (20–65 years)^5^ are insured through the statutory pension insurance (GRV: Gesetzliche Rentenversicherung), which at retirement provides a monthly pension that closely relates to the sum of earnings subject to compulsory insurance from contribution periods. For example, if earnings in a given year coincide with the average earnings of all insured individuals in the same year (50 % of the national average), 1.0 (0.5) remuneration points are credited. An individual is vested in their pension plan after having contributed for 60 months. Pension credits can also be earned during non-contribution periods for a limited time period for the following reasons (i) sickness, rehabilitation, higher education; (ii) military service or detention for political reasons; (iii) parenting or caring for family members, if this care required the individual to withdraw from the labour market; and (iv) during spells of unemployment while receiving unemployment benefits. The statutory pension insurance has different redistributive elements that explicitly and implicitly favour women during non-contributory periods (e.g. pregnancy, maternity, or parental leave). The most relevant one is parental leave. The person who takes responsibility for child-rearing (this defaults to mothers unless registered otherwise) gains 3 (2) earning points in the statutory pension insurance for children born after (before) 1992, independent of the person’s previous labour income. As women typically earn less than men, they usually profit more from these periods than men. For women who did not participate in the labour market before pregnancy, this benefit alone amounts to 95.67 (287.01) euros a month for one (three) child (children) in 2019 compared to an average of 890 euros gross pension for all retirees in Germany in the statutory pension insurance. In addition, pension entitlements of mothers with low earnings, for instance, from part-time work, can be topped up during periods of child-rearing (Frericks et al., 2008).

*Civil servant pension insurance* Roughly, 5% of working people in Germany are civil servants. The pension provided through civil servant pension insurance depends on the overall tenure and average salaries in the last position the individual held as a civil servant for at least two years. Each year of full time-service awards 0.01793375 replacement points up to a 0.7175 maximum. It is possible to receive both a statuary pension and a civil servant pension, although deductions apply. For child-rearing (parenting) periods, a supplement is granted comparable to the one in the statutory pension insurance.

*Pension insurance for the liberal professions* Liberal professions have a separate but compulsory pension scheme according to the laws of the Laender for about 85 liberal professions, including architects, chartered accountants, dentists, lawyers, notaries, pharmacists, physicians, and psychotherapists. These schemes provide old-age pensions, disability benefits, and survivors’ benefits. Entitlements are highly individual and are difficult to determine by simple rules. Liberal professions comprise roughly 3.5% of the workforce. Members of the liberal professions pension scheme can also apply for a child-rearing supplement from the statutory pension insurance and thus profit from this redistributive element.

*Second Pillar: Occupational Pension Schemes*

Occupational pension insurance is provided by companies to their employees on a voluntary basis. There are at least five different pensions plans in Germany. They comprise defined benefit (DB) plans, defined contribution (DC) plans, and also contributions with a minimum benefit. In 2019, about 54% of all employees subject to social security contributions had entitlements from occupational pension schemes (BMAS, 2021). Caregiver credits were only granted to employees in the public sector.^6^

In 2019, among retired individuals aged 65 and older, almost 90% received statutory pensions, 26% occupational pensions, only 5% civil servant pensions, and roughly 1% liberal profession pensions. In all pension schemes, gross rents for men are significantly higher than for women (see Table 1).

**Table 1:** Average pensions per month and share of persons with own pensions aged 65 and over (2019, amounts in Euros)

	All		Women		Men	
	Gross	Share	Gross	Share	Gross	Share
		(in %)		(in %)		(in %)
Statutory pensions	1.082	89	833	90	1.409	87
Civil servant	3.127	5	2.701	3	3.283	10
Liberal profession	2.163	1	1.659	1	2.378	2
Occupational pension	503	26	290	20	663	34
Occupational pension (public)	352	12	280	13	461	11

*Source:* BMAS (2020) Table B.2.1

## Data

We use the 2012 and 2013 waves of the Socio-Economic Panel (SOEP) (Goebel et al., 2019), which is an ongoing longitudinal survey of individuals living in private households in Germany. The 2012 wave includes the wealth module, which provides information on ten different asset and debt components for each adult in the household separately. These include property wealth (and associated debt), building loan contracts, financial assets (e.g. savings accounts, bonds, shares, or investments),^7^ private insurance policies, collectibles (in the form of gold, jewellery, coins, or valuable collections, etc.), net business assets (gross business assets minus debts) and on the debt side, consumer credits and mortgages. For wealth components that are held jointly, respondents are asked to state their individual share. In 2013, SOEP respondents were asked for the first time to report current pension entitlements based on the official annual information provided by their insurer for the year 2012. Using this information, pension wealth can be calculated based on the so-called “accrual method” (see (Wolff, 2015)) as the expected capitalised value of entitlements. Our primary dependent variable is augmented wealth, the sum of pension wealth and net wealth, which is the sum of assets minus total debts. Besides wealth and pension information, we use individual characteristics and information about the employment history, which is described in Appendix A.

The focus of our sample is the working-age non-retired population aged 25 to 60.^8^ Following (Sierminska et al., 2019), we top- and bottom-code wealth variables at 99.9% and 0.1%, respectively. Missing values are corrected with multiple imputation techniques (see (Grabka & Westermeier, 2015)).

## Framework and Empirical Strategy

We introduce the concept of augmented wealth into the standard framework for examining differences in wealth accumulation. Augmented wealth (*AW*) is the sum of net worth and pension wealth.

1 $$\begin{aligned} AW_{t+1}=NW_{t+1}+PW_{t+1} \end{aligned}$$

Net worth in period $t+1$ ($NW_{t+1}$) is the sum of assets (less debt) and income less consumption in period *t* augmented by the return on investments. In other words,

2 $$\begin{aligned} NW_{t+1}=(1+r)(NW_t+Y_t-C_t) \end{aligned}$$

where in period *t*, assets are $NW_t$, income $Y_t$, consumption $C_t$, and return on investments *r*, besides interest and dividends *r* also includes a change in the value of assets.

The literature provides evidence of gender differences in labour market attachment, income, risk preference, and household structure, which affect asset and wealth accumulation. Differences in income, however, affect both private wealth accumulation and pension entitlements directly since the latter are determined by years in the labour market and the wage level. (See Sect. 2.) Pension wealth (*PW*) is the sum of all present values of pensions entitlements ($PV_p$) (Bönke et al., 2019) and is calculated using the "accrual method" discussed in Wolff (2015).^9^

3 $$\begin{aligned} PW_{t+1}=\sum ^{}_{p} PV_p=\sum ^{}_{p}\sum ^{T-a}_{t=0} s_{a,t} \frac{1}{(1+i)^t} pension^p _t \end{aligned}$$

where $s_{a,t}$ is the probability of a person of age *a* in year 2012 surviving until year *t*; $T - a$ is the remaining maximum lifespan differentiated by sex and birth cohort provided by official statistics; *i* is the constant discount rate^10^ and $pension_t^p$ is the pension entitlement from pension scheme *p*.

When comparing average gross pension entitlements collected by SOEP with information from the statutory public pension insurance and occupational pension schemes,^11^ a high overlap is observed (see (Bönke et al., 2019)). We follow the previous literature on the determinants of wealth distributions by gender in our analysis (Sierminska et al., 2019) and define employment types (experience in years full-time and part-time employment), current occupation, industry, size of the company, education level, presence of children in the household and pension entitlement types (Frick & Grabka, 2013).

Our empirical strategy is to first decompose the wealth gap using the Oaxaca-Blinder (OB) method (Oaxaca, 1973; Blinder, 1973) at the mean. The specification for the decomposition is as follows:

4 $$\begin{aligned} \Delta _{\overline{x}}= (\overline{X}^M-\overline{X}^F) \hat{ \vartheta }^M +\overline{X}^M (\hat{ \vartheta }^M -\hat{ \vartheta }^F) \end{aligned}$$

The first component captures the average wealth differences due to characteristics ("explained effect") or endowments, and the second term captures the differences due to coefficients ("estimated effects") or returns to endowments.

Additionally, for the detailed decomposition of the gender wealth gaps across the wealth distribution, we use the technique introduced by Firpo, Fortin, and Lemieux (FFL) (2009). The FFL decomposition examines differences across the wealth distribution by allowing differences between distributions to be decomposed. This method relies on the estimation of a regression where the dependent variable is replaced by a recentred influence function (RIF) and can be applied in a similar way as the OB decomposition to any distributional statistic.

The FFL specification for the wealth gap is as follows:

5 $$\begin{aligned} \Delta _{Q\tau }= (\overline{X}^M-\overline{X}^F) \hat{ \vartheta }^M_{Q\tau } +\overline{X}^M (\hat{ \vartheta }^M_{Q\tau } -\hat{ \vartheta }^F_{Q\tau }) \end{aligned}$$

where $\Delta _{Q\tau }$ refers to differences in quantile $\tau$; $\overline{X}^M$ and $\overline{X}^F$ are average observed characteristics; $\hat{ \vartheta }^{M,F}_{Q\tau }$ are coefficients obtained from the regression of the RIF variables of quantile $Q\tau$ on the set of variables for men and women.

The first term refers to the effect on the gap between distributions caused by differences in characteristics ("explained" portion) and the second term can be interpreted as differences in returns to those characteristics of each explanatory factor ("unexplained" portion).

In the decomposition of the wealth equation, the determinants include individual demographic characteristics, labour market characteristics, and an indicator for pension types (Appendix A). For the FFL decomposition, we focus on the 25th, 50th, and 90th percentiles.^12^

## Descriptive Statistics

Table 2 provides a descriptive summary of mean and median wealth levels by gender. For all wealth components, men exhibit higher values. The mean difference in net wealth holdings for our sample between men and women of working age is close to 31,000 euros, a smaller difference than the one found in Sierminska et al. (2019) for a similar data sample that includes older individuals. At the mean, including pension wealth in the wealth measure increases the mean gender wealth difference to almost 45,000 euros (augmented wealth). The gender wealth gap, measured as the mean difference between male and female wealth as a proportion of male wealth, is thus reduced from 35 to 28%. This is due to the smaller gender wealth gap (20%) in pension wealth. On average, the bulk of pension wealth for both men and women consists of statutory pension wealth, which also has the lowest proportionate wealth gap (at 11%) resulting from the previously described redistributive elements of the statutory public pension scheme. Given that no redistribution takes place in occupational pension schemes, the unconditional wealth gap is 45% in civil servant pension wealth and 38% in occupational pension wealth at the mean and thus higher than for net worth.

At the median, the effects exhibit a similar pattern of smaller magnitude, with a gender gap of 10,000 euros in net wealth and 20,142 euros in augmented wealth due to the 11,186 euros gap in pension wealth (5,463 euros for statutory pensions). The median wealth for the remaining pension types is zero. Inequality indicators in the form of 1/2 the square of the coefficient of variation are in Appendix Table 12, indicating that the distribution of all wealth components suggests that pension wealth is more unequal among men. Additionally, inequality is much higher within the groups of men and women than between these groups.

**Table 2:** Summary wealth and pension wealth by gender

	Male (Mean)	Female (Mean)	Diff.	Gap (M-F)/M	Male (Median)	Female (Median)	Diff.
Net wealth	89,276	58,288	30,988	0.35	22,000	12,000	10,000
Augmented wealth	159,377	114,110	45,268	0.28	84,851	64,709	20,142
Pension wealth	69,873	55,658	14,215	0.20	44,751	33,566	11,186
Statutory	48,289	42,914	5375	0.11	32,573	27,110	5463
Civil	9749	5384	4365	0.45	0	0	0
Occupational	11,835	7360	4475	0.38	0	0	0

Sample SOEPv30 2012 and 2013, individuals between the ages of 25 and 60. Sample size 8894, with 4047 males and 4847 females. Means were calculated using the multiply imputed wealth data and sample weights. Differences in means are male mean minus female mean. Augmented wealth is the sum of net wealth plus the present value of total pensions. Pension wealth is the sum of statutory, civil, and occupational pension wealth that each individual has. The wealth gap is the ratio of male female mean difference over mean male wealth ((male-female)/male). All differences are statistically significant with $p<0.001$ except for occupational pensions, which is significant with $p<0.01$. All wealth variables are top- and bottom-coded at 99.9 and 0.1 per cent, respectively

The demographic characteristics for men, women, and the whole sample are presented in Tables 3 and 13 . On average, women are slightly younger and are more likely to be immigrants, to have more children, and to have a child 16 or younger living in their household. Compared to men, more women are married, divorced, or widowed. Men, in contrast, are more likely to be cohabiting or single. Men are also more likely to be from East Germany. In terms of education, most of the individuals in our sample have lower vocational education, with both women and men equally likely to be in this category.

**Table 3:** Descriptive statistics sample means for male, females, and total sample

	Male	Female	Total
Age	43.50	43.32	43.41
Immigrant	0.08	0.12	0.10
East	0.21	0.20	0.20
Number of children	1.25	1.49	1.38
Married	0.52	0.55	0.54
Cohabiting	0.12	0.10	0.11
Single	0.24	0.18	0.21
Divorced/separated	0.12	0.14	0.13
Widowed	0.00	0.02	0.01
*Education*			
Low education	0.09	0.10	0.10
Lower vocational	0.50	0.50	0.50
Upper vocational	0.15	0.15	0.15
University	0.24	0.23	0.23
*Occupation*			
Not employed	0.08	0.18	0.13
Trainee	0.03	0.02	0.03
Self-employed	0.10	0.07	0.08
White collar	0.43	0.54	0.49
Blue collar	0.29	0.14	0.21
Civil servant low	0.02	0.01	0.02
Civil servant high	0.04	0.03	0.04
*Experience, in years*			
Experience, years full-time	18.03	10.97	14.31
Experience, years part-time	0.98	5.16	3.18
Experience, years unemployed	1.14	1.17	1.15
*Pension rights*			
Has statutory pensions	0.84	0.88	0.86
Has civil servant pension	0.08	0.06	0.07
Has occupational pension	0.28	0.27	0.27
Observations	4047	4847	8894

*Source:* SOEPv30 2012 and 2013. The sample includes non-retired individuals between 25 and 60 years old. Sample weights are used. Variables are described in Appendix A.

Labour market differences between men and women are more noticeable than demographic differences. In terms of occupations, men are more likely than women to be self-employed, blue-collar workers, or civil servants (both high and low level). Women are more likely to hold white-collar jobs than men and are also more likely not to be employed.

Labour market experience is measured as the total years spent either in full-time or part-time employment. On average, men have 18 years of full-time working experience and slightly less than a year of part-time experience. Women have, on average, just 11 years of full-time working experience but five years of part-time experience. These differences in experience are important when explaining both the net wealth and augmented wealth gender gap.^13^

In our sample, women have a higher probability than men of having accumulated wealth from statutory pension rights (88% vs 84%), which can be explained by a higher share of self-employed men who are not bound to the statutory scheme. For occupational pensions, we observe no relevant differences between women and men, as about 27% of both groups hold these pension types. On the other hand, civil servant pensions are more common among men, with a share of 8% compared to 6% for women.

Table 4 shows the distribution of wealth components by net wealth deciles and shares of components of augmented wealth for women and men. Net worth contributes on average 56% to augmented wealth for men and 51% for women, while pension wealth contributes 44% and 49%, respectively. Statutory pensions play a diminishing role throughout the distribution. Pensions are the only contributor to augmented wealth in the second decile. Across all deciles, men have higher levels of wealth (of all types) except in the sixth, seventh and eighth decile of net worth, where the reverse is true. Statutory pension shares for these deciles play a larger role for women than for men.

**Table 4:** Distribution of wealth by net wealth deciles for males and females

Decile	Mean (in Euros)				Share of augmented wealth(%)			
	Net wealth	Total pension	Augmented wealth	Statutory pension	NW	TP	SP	CP
*Male*								
1	−17,076	49,229	31,863	38,125	−53.59	154.50	119.66	17.98
2	−58	39,730	39,671	35,644	−0.15	100.15	89.85	9.14
3	475	37,299	37,774	32,786	1.26	98.74	86.79	11.75
4	3345	34,092	37,995	30,028	8.80	89.73	79.03	8.13
5	10,860	51,173	62,033	38,616	17.51	82.49	62.25	8.64
6	24,232	65,053	91,804	46,645	26.40	70.86	50.81	13.16
7	47,501	73,380	120,881	52,132	39.30	60.70	43.13	10,00
8	84,736	97,170	181,906	60,756	46.58	53.42	33.40	11.48
9	143,091	104,129	247,219	64,104	57.88	42.12	25.93	7.58
10	485,629	121,311	606,514	70,632	80.07	20.00	11.65	4.34
Overall	89,276	69,873	159,377	48,289	56.02	43.84	30.30	7.43
*Female*								
1	−14,332	42,636	28,648	35,526	−50.03	148.83	124.01	20.16
2	−50	38,816	38,766	35,896	−0.13	100.13	92.60	5.86
3	379	36,892	37,270	33,830	1.02	98.98	90.77	5.87
4	3402	34,584	37,986	29,266	8.96	91.04	77.04	10.16
5	10,485	45,431	55,916	35,084	18.75	81.25	62.74	11.64
6	24,400	51,858	76,258	38,232	32.00	68.00	50.13	10.67
7	48,144	64,522	112,666	50,223	42.73	57.27	44.58	9.42
8	85,142	76,401	161,543	53,895	52.71	47.29	33.36	5.88
9	142,761	87,050	230,550	62,158	61.92	37.76	26.96	4.92
10	363,886	92,380	457,023	62,262	79.62	20.21	13.62	3.67
Overall	58,288	55,658	114,110	42,914	51.08	48.78	37.61	6.45

*Source:* SOEPv30 2012 and 2013, weighted results. The sample includes non-retired individuals between 25 and 60 years old. NW, TP, SP, and CP refer to net wealth, total pension, statutory pension, and occupational pension as shares of augmented wealth. Sample size is 8,894, 4,047 for males and 4,847 for females.

## Decomposition Results

### Gender Wealth Gap Decomposition

We use two different approaches to decompose the wealth gap. First, we estimate Eq. 4, a standard Oaxaca–Blinder (OB) decomposition in the literature.^14^ This decomposition concentrates on the mean of the wealth variables, and we estimate it separately for net wealth, augmented wealth, and pension wealth. The specifications include the complete set of control variables listed in Appendix A. Tables 5 and 6 show the estimated gender gap in percentages in terms of men’s wealth, estimated wealth for men and women, the difference, and the explained and unexplained portions of the difference for the full sample and by age cohorts. The full OB decomposition of the gender wealth gap is in Appendix Table 16. The discussion focuses on statistically significant factors. Accounting for pension wealth in the measure of net worth decreases the gender wealth gap from 36.2% in terms of men’s net wealth to 30.1%. In other words, the wealth gap narrows after accounting for pension wealth due to the smaller relative gap of 21.3% in this type of wealth. At the same time, the absolute difference in wealth increases.

Additionally, in Tables 5 and 6 , we compare the results over *age cohorts*. There, we find substantially lower wealth levels (independent of the type of wealth) among younger age cohorts. For example, net worth amounts to 30,000 euros for men in the youngest cohort, increases to 114,000 euros in the middle-age cohort, and reaches its highest value of 164,000 euros in the oldest cohort (49–60 years). A similar increasing pattern is observed for women. In younger cohorts, women participate more actively in the labour market (Destatis, 2020).^15^ Thus, given that labour market variables play a major role in explaining the gap in pension wealth, the gap is expected to be smaller for the younger cohort.^16^ The results in Tables 5 and 6 by age cohort indicate that the gap in net wealth is the largest, at 43.3%, for 25–36-year-old individuals, while the gap in pension wealth is the smallest for this group (4.6%). This small gap in pension wealth points to the relatively similar labour force participation rates of men and women in younger cohorts. The relatively large absolute gap in net worth among older individuals stems, among other things, from the fact that gender differences in labour market outcomes have accumulated and been magnified up to this later point in the life course. Since the majority of wealth is held by the older population, the gaps for those over 48 coincide largely with those for the whole population.

For the sample as a whole, differences in characteristics explain around one quarter of the gender difference in net wealth, while the unexplained component—the returns to those characteristics—account for around three quarters of the difference. Appendix Table 16 indicates that the most important components of the explained portion of the gap are differences in self-employment, work experience, having a white-collar occupation, company size, being divorced, and not being employed. The differences in working part-time, not being employed, having a white collar occupation and the size of a company favour women and help close the gap. In contrast, differences in being self-employed, being divorced, years worked full-time, and being unemployed favour men. The characteristics that contribute positively to the unexplained component (the returns) are: having attended university and being self-employed. The characteristic that contributes negatively to the unexplained component is being from East Germany, which reflects the historically poorer opportunities for wealth accumulation before reunification as well as the generally poorer labour market situation and the associated lower wage level in this region. In the augmented wealth decomposition, the share of the explained portion increases to almost half, given that pension wealth is highly correlated to lifetime earnings. The main differences from the net wealth decomposition resulting from characteristics are differences in industries, which favour men.

The OB decomposition of the pension wealth gap shows that differences are almost entirely due to differences in characteristics. Differences in having children, self-employment, being a white-collar worker, and years of experience as a part-time employee help close the gap in pension wealth for women. The coefficient on the size of the company becomes positive and contributes to the increase in the pension wealth gap.

**Table 5:** Oaxaca–Blinder decomposition at means of the gender wealth gap, pension wealth, and augmented wealth for the whole sample and youngest age cohort

	All Ages 25–60			Age cohort 25–36		
	Net wealth	Augmented wealth	Pension wealth	Net wealth	Augmented wealth	Pension wealth
Gap(%)	36.2%	30.1%	21.3%	43.3%	29.4%	4.56%
Male	114,118.9***	194,278.4***	79,994.0***	30,205.1***	46,695.3***	16,490.2***
	(4928.5)	(5344.0)	(1486.0)	(5781.1)	(5834.6)	(761.5)
Female	72,699.3***	135,788.2***	62,932.0***	17,104.9***	32,926.5***	15,737.3***
	(2273.8)	(2711.0)	(1061.6)	(1237.5)	(1439.7)	(615.9)
Difference	41,419.6***	58,490.2***	17,062.0***	13,100.3*	13,768.8*	752.9
	(5427.7)	(5992.4)	(1826.3)	(5912.0)	(6009.7)	(979.4)
Explained	9272.1*	27,391.1***	18,248.5***	−567.7	−741.0	−190.7
	(3790.7)	(4470.3)	(1701.9)	(3671.1)	(3829.1)	(915.8)
Unexplained	32,147.5***	31,099.1***	−1,186.5	13,667.9	14,509.7	943.6
	(6202.8)	(6618.3)	(1756.4)	(7850.9)	(7904.7)	(1004.8)
Observations	8894	8894	8894	2205	2205	2205

Estimated using the SOEPv30 2012 and 2013 sample including non-retired individuals ages between 25 and 60 and sample weights. Percentages in terms of males. Decomposition estimated using the full set of controls described in Appendix A. The wealth gap is calculated in terms of males.

**Table 6:** Oaxaca–Blinder decomposition at means of the gender wealth gap, pension wealth, and augmented wealth by age cohort

	Age cohort 37–48			Age cohort 49–60		
	Net wealth	Augmented wealth	Pension wealth	Net wealth	Augmented wealth	Pension wealth
Gap(%)	40.3%	33.7%	20.1%	32.3%	27.4%	21.7%
Male	114,229.5***	171,563.4***	57,078.0***	163,943.8***	300,855.0***	136,722.1***
	(8598.0)	(8853.0)	(1511.9)	(8724.7)	(9331.9)	(2750.3)
Female	68,152.2***	113,716.0***	45,558.9***	110,958.8***	218,269.4***	106,978.7***
	(3861.9)	(4033.6)	(998.7)	(4290.6)	(5025.9)	(2049.3)
Difference	46,077.3***	57,847.4***	11,519.1***	52,985.0***	82,585.6***	29,743.4***
	(9425.5)	(9728.6)	(1812.0)	(9722.7)	(10,599.3)	(3429.9)
Explained	14,725.9*	29,150.7***	14,474.6***	11,736.4	43,373.5***	31,762.2***
	(6638.7)	(6932.0)	(1672.1)	(7691.9)	(8704.7)	(3392.8)
Unexplained	31,351.4**	28,696.8*	−2955.4	41,248.6***	39,212.1**	−2,018.8
	(11,689.2)	(12,036.8)	(2270.4)	(12,263.0)	(13,260.4)	(3942.8)
Observations	3059	3059	3059	3630	3630	3630

Estimated using the SOEPv30 2012 and 2013 sample including non-retired individuals ages between 25 and 60 and sample weights. Percentages in terms of males. Decomposition estimated using the full set of controls described in Appendix A. The wealth gap is calculated in terms of males.

The second approach we utilise to estimate the gender wealth gap is the detailed decomposition for the whole distribution. We estimate Eq. (5) using the FFL recentred influence function decomposition method (RIF) for the 25th, 50th, and 90th percentiles. The results from these are summarised in Table 7. The complete results can be found in Appendix Tables 17,  18, and  19. These estimations also include the full set of control variables. The largest gap is at the bottom of the net wealth distribution, at the 25th percentile (90%), narrowing down to 37.8% at the median and 26.5% at the 90th percentile. In contrast to net wealth, the pension wealth distribution has a significantly smaller wealth gap at the bottom of the distribution of 10.1%, increasing to a somewhat wider gap of 21.2% at the median and 20.8% at the 90th percentile of the distribution. Thus, including pension wealth decreases the augmented wealth gap at the bottom of the distribution to 21.5% and 21.6% at the bottom 25th percentile and 50th percentile, respectively. But at the 90th percentile, the gender wealth gap remains at almost the same level as in net worth at 30%. This is where the smallest share of pension wealth constitutes augmented wealth (as per Table 4). This is partly due to an assessment ceiling in statutory pension insurance, which limits the influence of this component.

The differences in net wealth between men and women vary across the distribution, and so do the contributions of the explained and unexplained components of the decomposition. The overall differences in characteristics contribute positively to the gap across the distribution and explain over half of the differences in the net wealth distributions. The differences in returns, or the unexplained component, favour women with a negative contribution to the difference throughout the whole distribution.

At the bottom of the distribution, the unexplained components—which favour women substantially—contribute to reducing the gap in both net worth and augmented wealth. At the top of the distribution, however, the differences in characteristics account for most of the gap. The statistically significant returns that contribute negatively include not being employed and industry type. At the top, only the returns to being widowed and experience in part-time employment help to close the gap. Differences in characteristics that contribute to the gap include: self-employment (+), white collar occupations (-), industry (+25th, +50th), company size (-90th), being divorced (+25th, +50th), being widowed (+90th), experience working full-time (+), experience working part-time (+90th), and being unemployed (+20th, +50th). Thus, to further decrease the gap in characteristics, women would need to be self-employed, in more similar industries to men, not lose as a result of divorce or widowhood, and have similar experience working full-time and part-time.

**Table 7:** RIF-OAXACA decomposition of gender gap, population 25--60

	(1)	(2)	(3)
	Q25	Q50	Q90
*Net wealth*			
Gap(%)	90.3%	37.8%	26.5%
Male	1,755.92$$*$$	32,985.83$$***$$	261,051.92$$***$$
Female	171.11	20,514.79$$***$$	191,978.74$$***$$
Difference	1584.81	12,471.04$$***$$	69,073.18$$***$$
Explained	9,974.06$$***$$	25,674.91$$***$$	117,993.01$$***$$
Unexplained	−8,389.25$$***$$	−13,203.87$$***$$	−48,919.83$$**$$
*Augmented wealth*			
Gap(%)	21.5%	21.6%	30%
Male	35,606.59$$***$$	104,686.24$$***$$	441,057.15$$***$$
Female	27,941.02$$***$$	82,124.06$$***$$	308,558.83$$***$$
Difference	7,665.57$$***$$	22,562.18$$***$$	132,498.33$$***$$
Explained	23,249.64$$***$$	58,872.41$$***$$	163,094.04$$***$$
Unexplained	−15,584.07$$***$$	−36,310.23$$***$$	−30,595.72
*Pension wealth*			
Gap(%)	10.1%	21.2%	20.8%
Male	18,591.39$$***$$	52,141.44$$***$$	180,664.76$$***$$
Female	16,701.36$$***$$	41,046.49$$***$$	143,067.56$$***$$
Difference	1890.02$$*$$	11,094.95$$***$$	37,597.20$$***$$
Explained	7942.37$$***$$	19,013.44$$***$$	46,576.87$$***$$
Unexplained	−6,052.35$$***$$	−7918.48$$**$$	−8979.67

Estimated using the SOEPv30 2012 and 2013 sample with 8894 observations including non-retired individuals ages between 25 and 60 and sample weights, 8894 observations. Percentages in terms of males

$$*$$ $$p<0.05$$, $$**$$ $$p<0.01$$, $$***$$ $$p<0.001$$

### Decomposition by Pension Entitlements

Next, we study each of the pension entitlements distributions separately. Table 8 includes the decomposition estimates for Eq. (4) for each pension type. The mean decomposition includes the full set of control variables. Around 87% of our sample has some type of statutory pension wealth. There is an estimated 13.1% gender gap in statutory pensions, 32.7% in civil pensions, and 41.8% in occupational pensions. The relatively small gap in statutory pensions can be explained in large part by two aspects: first, the contribution ceiling, which limits the accumulation of earning points in the public scheme for high earners, and second, the aforementioned redistributive elements. Only, 8% of the individuals in our sample have civil servant pensions. Within the civil service, men often hold higher positions than women, so differences in characteristics explain almost all of the gap in this pension type. The biggest gender pension gap is in occupational pensions. Occupational pension wealth is positive for 30% of our sample. The gap of 41.8% has an explained and unexplained component that accounts for about half of the gap, both favouring men. Occupational pensions are typically provided by larger companies with higher earnings levels and in industries with a higher share of male workers. Additionally, there is no upper contribution ceiling that might reduce pension entitlements (and thus the gap).

**Table 8:** Oaxaca–Blinder decomposition at means of the statutory pension wealth gap, civil pension wealth, and occupational pension wealth

	(1)	(2)	(3)
	Statutory	Civil	Occupational
Gap(%)	13.1%	32.7%	41.8%
Male	53,253.9$$***$$	12,177.6$$***$$	14,562.5$$***$$
Female	46,268.0$$***$$	8190.0$$***$$	8474.0$$***$$
Difference	6,985.8$$***$$	3987.6$$***$$	6088.6$$***$$
Explained	11,126.8$$***$$	4002.3$$***$$	3119.4$$***$$
Unexplained	−4141.0$$***$$	−14.7	2969.1$$**$$
Observations	8894	8894	8894

Estimated using the SOEPv30 2012 and 2013 sample including non-retired individuals ages between 25 and 60. The statutory pension wealth decomposition includes the full set of control variables and can be found in Appendix A

$$*$$ $$p<0.05$$, $$**$$ $$p<0.01$$, $$***$$ $$p<0.001$$

Table 9 includes estimates of the RIF decomposition for statutory pension at the 25th, 50th and 90th percentile of its distribution. We focus on this pension, as it not only has the highest prevalence, but also quantitatively constitutes the largest component of pension wealth. At the 25th percentile, the gender wealth gap is in favour of women with a value of −7.4%. This result corresponds to the one by age group, as the wealth gap is generally smaller for younger people than for older ones due to similar employment histories of women and men at this stage and the impact of the redistributive element of pensions, which contributes more to women’s pension value at this stage.

**Table 9:** RIF-OAXACA decomposition of statutory pension wealth gap

	Statutory Q25	Statutory Q50	Statutory Q90
Gap(%)	−7.4%	16.5%	14.3%
Male	10,649.6$$***$$	38,144.4$$***$$	128,505.5$$***$$
Female	11,440.5$$***$$	31,846.0$$***$$	110,066.9$$***$$
Difference	−790.9	6298.5$$***$$	18,438.6$$***$$
Explained	2548.2$$*$$	14,344.3$$***$$	28,070.7$$***$$
Unexplained	−3339.1$$**$$	−8045.8$$***$$	−9632.0

Estimated using the SOEPv30 2012 and 2013 sample of 8894 observations including non-retired individuals ages between 25 and 60. The estimation includes the full set of controls and can be found in Appendix A

$$*$$ $$p<0.05$$, $$**$$ $$p<0.01$$, $$***$$ $$p<0.001$$

At the median, there is a gap of 16.5% due to emerging differences in characteristics favouring men. Here, the gender pay gap becomes more relevant in explaining the gap—and so do the returns to characteristics favouring women in statutory pension accumulation. At the 90th percentile, the gap is 14.3% and almost triples in absolute terms. The difference in characteristics in this case plays a large role. The returns, which reduce the difference, are smaller in percentage terms than at other points of the distribution.

### East and West Germany

As pointed out in the introduction, there are still pronounced economic, cultural, and normative differences between East and West Germany. These are also reflected in different wealth levels in Table 10. For example, the net worth for men is only about 58,810 euros in East Germany, while it is more than twice that in West Germany (132,416 euros). Average augmented wealth for men is also almost 100,000 euros higher in the western part of the country. For pension wealth, however, the advantage is much smaller for people living in the West. As discussed in sect. 2, the disadvantages faced by women in the West, due to the still prevailing norms of the male breadwinner model, result in a gender private wealth gap of 37.6% compared to only 29.8% in the East. While the gender gap decreases only slightly to 33.8% for augmented wealth in West Germany, it decreases to 10.8% in East Germany due to the much smaller gap in pension wealth in that part of the country. In East Germany, women hold 65,220 euros in pension wealth and men hold 60,883 euros. In West Germany, in contrast, the pension wealth gap is about 27.9%, with men holding 24,105 euros more in pension wealth, due to the more pronounced gender wage gap in that region and the higher prevalence of occupational pensions among men. Although women in the East have succeeded in narrowing the augmented wealth gap more than women in the West, East German women hold the lowest augmented wealth levels of all four groups under consideration. The relevance of the explained component also differs between the two regions. While observed characteristics contribute little to explaining the differences in East Germany, they explain almost 50% or more of the differences in West Germany, which means that if women had more similar characteristics to men in the West, the gaps would be much smaller.

**Table 10:** Oaxaca–Blinder decomposition at means of the gender wealth gap, augmented wealth, and pension wealth by region

	East Germany			West Germany		
	Net wealth	Augmented wealth	Pension wealth	Net wealth	Augmented wealth	Pension wealth
Gap	29.8%	10.8%	−7.1%	37.6%	33.8%	27.9%
Male	58,810.2$$***$$	119,633.0$$***$$	60,882.8$$***$$	132,415.7$$***$$	218,971.9$$***$$	86,316.2$$***$$
	(4474.7)	(5291.9)	(2243.6)	(6355.1)	(6834.6)	(1818.7)
Female	41,277.7$$***$$	106,701.8$$***$$	65,219.7$$***$$	82,596.3$$***$$	144,949.7$$***$$	62,211.4$$***$$
	(2733.6)	(4304.7)	(2345.8)	(2843.9)	(3282.7)	(1184.4)
Difference	17,532.5$$***$$	12,931.2	−4336.9	49,819.4$$***$$	74,022.2$$***$$	24,104.8$$***$$
	(5243.6)	(6821.7)	(3246.0)	(6962.4)	(7582.1)	(2170.4)
Explained	1701.1	6653.7	4810.6	24,192.0$$***$$	51,720.1$$***$$	27,564.1$$***$$
	(3110.6)	(4671.9)	(2581.7)	(4447.9)	(5135.6)	(1851.6)
Unexplained	15,831.4$$**$$	6277.5	−9147.5$$***$$	25,627.4$$***$$	22,302.1$$***$$	−3459.3
	(5524.4)	(6876.3)	(2690.6)	(6245.5)	(6667.1)	(1828.4)
Observations	2167	2167	2167	6727	6727	6727

Estimated using the SOEPv30 2012 and 2013 sample including non-retired individuals ages between 25 and 60 and sample weights. Percentages in terms of males. Decomposition estimated using the full set of controls described in Appendix A. The wealth gap is calculated in terms of males.

### Robustness Checks

To check the robustness of our results, we estimate the gender wealth gap decompositions in restricted samples. First, we exclude self-employed individuals from our analysis because contributions to statutory and occupational pensions are not compulsory for the self-employed (only for certain occupations). Thus, we expect the relative gap to be smaller. We estimate the FFL recentred influence function decomposition method for the 25th, 50th, and 90th percentile for this sub-sample. Table 22 shows that excluding the self-employed from the sample results in slightly smaller net wealth and augmented wealth gaps. These reductions are consistent with the explained portion in the case of the whole sample, where self-employment makes a significant positive contribution to the gap. For pension wealth, the gap is now stable at 16 to 19% over the whole distribution. Excluding the self-employed leads to a more homogeneous population, where differences in earning levels between sexes play the main role in the gap. The unexplained portion helps reduce the gap.

**Table 11:** Oaxaca–Blinder mean decomposition RIF-OAXACA median wealth gaps, individuals without children

OB	(Mean)		
	Net wealth	Augmented wealth	Pension wealth
Gap(%)	24.1%	11.9%	−5.6%
Male	79,746.0$$***$$	133,077.4$$***$$	53,374.3$$***$$
Female	60,560.3$$***$$	117,208.3$$***$$	56,387.4$$***$$
Difference	19,185.7$*$	15,869.2	−3013.1
Explained	−4718.0	−9461.7	−4490.1
Unexplained	23,903.7$*$	25,330.9$*$	1477.1

RIF-OB	(Q50)		
	Net wealth	Augmented wealth	Pension wealth
Gap(%)	0.7%	−10.1%	−8.2%
Male	10,527.2$$***$$	48,398.8$$***$$	26,550.0$$***$$
Female	10,448.2$$***$$	53,276.3$$***$$	28,726.3$$***$$
Difference	78.9	−4877.5	−2176.4
Explained	18.3	525.6	−595.9
Unexplained	60.6	−5403.1	−1580.4
Observations	2225	2225	2225

Estimated using the SOEPv30 2012 and 2013 sample including non-retired individuals without children ages between 25 and 60 and sample weights. Percentages in terms of males. Decomposition estimated using the full set of controls described in Appendix A

* *p* < 0.05, ** *p*< 0.01, ***p < 0.001

Next, we restrict the sub-sample to adults without children in order to focus on individuals who have no career interruptions due to child-rearing. Table 11 shows the estimation results for the mean and median decomposition for all wealth variables. In this case, the differences are only statistically significant for the net wealth mean decomposition. Net worth is significantly lower for individuals without children. This is partly because some individuals are single, who do not profit from the economies of scale that arise from cohabitation and also do not profit from joint taxation of married couples. Additionally, individuals without children are often younger than the sample as a whole and are thus still at the beginning of their working life. All this leads to a significantly lower gap in net wealth. At the mean, the relative gap is only about 24% compared to 36% for the total population including those with children. For pension wealth, the gap is negative—although not statistically significant—that is, men show lower levels of pension wealth than women. This is in line with previous research showing that women without children perform better in the German labour market, while motherhood entails significant risks for both a career and pension entitlements (Schrenker & Zucco, 2020). As a result, the gap in augmented wealth is strongly reduced for those without children and is even negative at the median at −10% compared to 21.6% for the total population.^17^

## Limitations

The analyses presented here face some limitations. First, these concern the availability of information on pension entitlements. So far, this information has been collected in SOEP in 2013 for the 2012 reference year. This means that the effect of the maternity pension introduced in 2014 is not taken into account. It stipulates that mothers or fathers are credited with an additional year of child-raising time for children born before 1992. However, one additional pension point only corresponds to an additional monthly pension entitlement of 34.19 euros in 2020 in West Germany and therefore has a limited effect. Besides this, the continued rise in women’s labour force participation in Germany is likely to have had a positive impact on the gender gap in pension wealth and will be visible in more recent data. Between 2012 and 2019 alone, women’s labour force participation increased by 4.8 percentage points according to the Federal Statistical Office.^18^

Another data-related limitation concerns the under-representation of multimillionaires and billionaires in SOEP (see (Westermeier & Grabka, 2015) and their potential under-reporting of assets (Davies, 2009)). The fact that there are very few women among the top 1,000 richest individuals in Germany, as shown by the “rich list” of the German Manager-Magazin, indicates that our estimates of the wealth gap can be treated as a lower bound of the real gap at the top. This affects the OB decomposition but not our preferred RIF decomposition, where we look at the 90th percentile and not at the very top of the distribution.^19^ Moreover, our measure of net worth consisting of ten different asset components does not include, for instance, the value of vehicles or student loans (both collected in SOEP for the first time in 2017). Although there are no gender-specific differences in the spread of student loans, men have an almost 20% higher probability of owning vehicles compared to women and thus slightly underestimating the gap for private wealth.

One final limitation concerns pension entitlements for liberal professions, as they are not collected for the working-age population in SOEP but only for retirees. Although the level of entitlements among those who are eligible is above average compared to the statutory pension scheme, the share of recipients is low at around one percent. Thus, the overall effect of this omission should be negligible. It should also be noted that the gender pension gap for beneficiaries is around ten percentage points lower than in the statutory pension insurance (see Table 1).

## Conclusion

We extend the study of the gender wealth gap by including pension wealth in the standard measure of net worth. For this purpose, we use detailed individual data on personal wealth and pension entitlements of the working-age population from the 2012 and 2013 waves of the German SOEP. The unconditional gender wealth gap increases in levels from an average of 31,000 euros to 45,000 euros when pension wealth is included, while the relative gap decreases from 35 to 28%. We take two approaches to estimate a conditional gender wealth gap. First, we estimate an OB decomposition at the mean and find that including pension wealth decreases the relative gender wealth gap from about 36.2 to 30.1%.

The second approach we take is to estimate a RIF decomposition following Firpo et al. (2009) at the 25th, 50th, and 90th percentile of the wealth distributions. The net wealth gender gap is 90.3%, 37.8%, and 26.5%, respectively. The estimates of the FFL decomposition show that including pension wealth closes the gap at the bottom 25th and 50th percentiles but changes almost nothing at the 90th percentile. Pension wealth has much lower gaps of 10.1%, 21.2%, and 20.8% for each of the studied percentiles. Thus, the augmented wealth gaps are reduced, and decomposition estimates show that women have 21.5%, 21.6%, and 30% less augmented wealth than men, respectively. Differences in characteristics play a dominant role in explaining the gap for all three wealth concepts and across their distributions, always favouring men. The most important components for the explained portion are differences in self-employment, work experience, having a white-collar occupation, company size, being divorced, and not being employed. This means that if women had the same characteristics as men, their wealth would be significantly greater and the gap correspondingly smaller.

Additionally, we estimate a decomposition for each pension type separately. The mean decomposition shows that women have a small disadvantage in statutory pension wealth. This results from a contribution ceiling in the statutory pension scheme and redistributive elements that compensate for non-working periods such as caregiver credits. Civil servant pensions show a gap of 32.7% at the mean, which is mainly explained by differences in characteristics. Of all pension wealth components, occupational pensions have the largest gap of 41.8% at the mean. For the RIF decomposition, there is no statically significant difference at the bottom 25th percentile of statutory pension wealth, and a 16.5% and 14.3% wealth gap at the 50th and 90th percentiles, respectively. A separate analysis for East and West Germany—two regions still exhibiting cultural, normative, and economic differences—shows the gender gap in net worth and in augmented wealth to be much lower in the East than in the West. The smaller gap in augmented wealth is the result of a negative gender gap in pension wealth (favouring women) in the East. But at the same time, the wealth levels in the East are also significantly lower than in the West.

This is, as far as we know, the first paper to show the impact of pension wealth when analysing the gender gap in net worth in Germany. As the German pension system is predominantly oriented towards the equivalence principle, gender-specific differences in the labour market translate directly into differences in pension wealth. Pension wealth is nevertheless more equally distributed between men and women than net worth. The implicit and explicit redistributive elements of the statutory pension scheme in Germany, which help those out of the labour market are largely responsible for this finding. However, these redistributive elements cannot fully reduce the various disadvantages faced by women when it comes to wealth creation in Germany. These consist of the very high gender pay gap of almost 20%, a lower employment rate of women, lower working hours, and concentration in occupations and sectors in which occupational pensions are provided less frequently. All these aspects lead to lower augmented wealth for women compared to men.

As it is unlikely that the German pension system will undergo a fundamental change towards, for example, a universal pension scheme, the question arises of how the gap in net worth and pension wealth can be narrowed. First of all, it is necessary to reduce the wage gap. As Frey (2021) shows, pay transparency tools can help to reduce this gap. In addition to increasing women’s working hours, the general conditions favouring the reconciliation of work and family life need to be improved, for example, by expanding all-day care facilities, especially for shift workers. To reduce the motherhood penalty, policymakers should consider changes in the tax regime. Instead of a joint taxation model, separate tax assessments could provide an impulse to increase women’s labour market participation. Ultimately, support programs are needed to ensure that young women choose to pursue training and education for better-paid jobs in order to reduce the gender pay gap. Future research should make cross-country comparisons to examine different pension systems and their impacts on the gender wealth gap. Universal pension systems, in particular, should be used for this purpose, as they compensate for income differences to a greater extent than purely income-dependent systems. In such systems, individuals are not penalised for their different life choices, especially when it comes to having children or doing care work.

## Appendix A

The full set of control variables includes the number of children (total number of births), number of children in the household (children 16 years or younger living in the household), immigrant (0–1 indicator for being born in Germany), East (0–1 indicator for living in East Germany), East/Female interaction (0–1 indicator to control for differential trends), education (secondary only (omitted category), lower vocational, upper vocational and university), employment status (not employed, trainee, self-employed), occupation (blue collar (omitted category), white collar, low and high-level civil servant), marital status (married (omitted category), cohabiting, single, divorced/separated, widowed), experience in years (full-time employment, part-time employment and being unemployed), and indicator for having pension rights (statuary, civil servant and occupational). Experience is expressed in calendar years and pension rights is an indicator of the individual having a positive amount of the present value of the corresponding pension. In a robustness check, we also control for age cohort, ages 25–36, 37–48, and 49–60 (omitted category).

We also include indicators for company size: no coworkers, small company (2–20 workers), medium company (20–200 workers, omitted category), and large (200 or more workers). Industry occupation indicators from NACE class 1.1 classifications: agriculture hunting and forestry(omitted), fishing, mining, manufacturing, electricity, gas and water supply, construction, wholesale and retail, hotels and restaurants, transportation storage and communication, financial intermediation, real estate, Public admin. and defence, education, health and social work, other community social and personal service activities, activities of households, and extraterritorial organisations and bodies.

See Tables 12, 13, 14, 15, 16, 17, 18, 19, 20, 21, 22 and 23.

**Table 12:** Inequality measures for augmented wealth components (1/2 CV)

	NW	PW	AW	SP	OP
GE(2)	4031	0728	1518	0572	6608
Male	4338	0699	1673	0572	6413
Female	2679	0732	1076	0562	6375
Within	4006	0722	1503	0570	6592
Between	0025	0006	0015	0002	0016

*Source:* SOEPv30 2012 and 2013. The sample includes non-retired individuals between 25 and 60 years old. NW, PW, AW, SP, and OP refer to net wealth, pension wealth, augmented wealth, statutory pension, and occupational pension, respectively

**Table 13:** Descriptive statistics sample means for male, females, and total sample

	Mean		
	Male	Female	Total
*Industry*			
Agriculture, hunting and forestry	0.01	0.01	0.01
Fishing	0.00	0.00	0.00
Mining and quarrying	0.00	0.00	0.00
Manufacturing	0.26	0.10	0.17
Electricity, gas and water	0.01	0.01	0.01
Construction	0.10	0.01	0.05
Wholesale and retail	0.07	0.11	0.09
Hotels and restaurants	0.01	0.03	0.02
Transp., storage and com.	0.06	0.03	0.04
Financial intermediation	0.03	0.03	0.03
Real estate	0.10	0.08	0.09
Public adm. and defence	0.07	0.06	0.06
Education	0.03	0.08	0.06
Health and social work	0.05	0.16	0.11
Other ser. act.	0.04	0.03	0.04
Activities of households	0.00	0.01	0.00
Extra-territorial org.	0.00	0.00	0.00
*Company size*			
No coworkers	0.05	0.04	0.04
Small company	0.18	0.20	0.19
Medium company	0.22	0.19	0.21
Large company	0.42	0.34	0.38
Observations	4047	4847	8894

*Source:* SOEPv30 2012 and 2013. The sample includes non-retired individuals between 25 and 60 years old. Sample weights are used. Variables are described in "Appendix A".

OLS regression estimates utilising the SOEPv30 2012 and 2013. The sample includes non-retired individuals between 25 and 60 years old and sample weights. Each column includes the estimates for indicated wealth variable as the dependent variable and the indicated gender. OLS regression estimated using the full set of controls described in Appendix A. Standard errors in parenthesis

**Table 14:** Determinants of wealth for overall population aged 25–60, by gender

	Net wealth		Augmented wealth		Pension wealth	
	Male	Female	Male	Female	Male	Female
Number of children	9713.5	1965.6	12,485.1$$*$$	4633.0	2551.2$$*$$	2741.8$$***$$
	(5,006.8)	(2360.0)	(5224.9)	(2579.7)	(1145.8)	(808.5)
Children in household	12,692.0	9783.9	8972.4	7922.4	−3356.0	−1921.6
	(12,469.4)	(5605.1)	(13,012.5)	(6127.0)	(2853.6)	(1920.3)
Immigrant	−47,639.5$$*$$	−41,464.8$$***$$	−57,901.5$$*$$	−47,180.6$$***$$	−10,120.1	−5627.8
	(22,601.7)	(9569.4)	(23,586.2)	(10,460.4)	(5172.3)	(3278.5)
East	−62,307.2$$***$$	−36,345.8$$***$$	−74,277.7$$***$$	−42,413.1$$***$$	−11,729.4$$***$$	−6,038.8$$**$$
	(11,077.0)	(5520.1)	(11,559.5)	(6034.1)	(2534.9)	(1891.2)
Age cohort 25−36	−32,954.5	−80,391.2$$***$$	−58,396.2$$*$$	−108,073.2$$***$$	−23,283.4$$***$$	−27,873.6$$***$$
	(26,043.5)	(9409.7)	(27,177.8)	(10,285.9)	(5959.9)	(3223.8)
Age Cohort 37−48	−15,769.1	−40,965.6$$***$$	−48,624.5$$**$$	−69,240.0$$***$$	−32,264.3$$***$$	−28,173.2$$***$$
	(15,657.7)	(6695.5)	(16,339.6)	(7319.0)	(3583.2)	(2293.9)
Lower vocational	18,834.5	16,033.8$*$	17,011.3	12,136.6	−2029.9	−3780.1
	(19,683.5)	(8019.1)	(20,540.8)	(8765.8)	(4504.5)	(2747.4)
Upper vocational	26,091.1	22,022.1$*$	25,925.8	17,934.2	−198.8	−4015.4
	(22,505.8)	(9306.7)	(23,486.1)	(10,173.3)	(5150.4)	(3188.5)
University	101,689.0$$***$$	48,873.3$$***$$	131,032.8$$***$$	53,628.4$$***$$	28,947.6$$***$$	4641.6
	(22,602.7)	(9168.5)	(23,587.1)	(10,022.2)	(5,172.5)	(3141.2)
Not employed	21,686.8	35,410.1$$***$$	21,390.3	36,086.5$$***$$	932.5	689.1
	(23,684.7)	(9268.0)	(24,716.3)	(10,131.0)	(5420.1)	(3175.3)
Trainee	42,009.9	34,774.2$$*$$	60,587.0	46,024.1$$*$$	19,943.4$$**$$	11,371.1$$*$$
	(33,062.2)	(16,640.2)	(34,502.2)	(18,189.6)	(7566.1)	(5701.0)
Self-employed	353,809.0$$***$$	120,893.9$$***$$	324,299.2$$***$$	101,639.2$$***$$	−26,962.8$$***$$	−18,810.7$$***$$
	(22,199.5)	(12,460.9)	(23,166.4)	(13,621.1)	(5080.3)	(4269.2)
White collar	22,958.1	26,352.7$$***$$	35,563.4$$**$$	30,058.0$$***$$	12,749.4$$***$$	3753.1
	(13,061.0)	(7278.6)	(13,629.9)	(7956.3)	(2988.9)	(2493.7)
Civil servant low	78,633.9	26,773.8	81,816.5	33,351.7	3101.0	7247.0
	(56,298.2)	(29,974.6)	(58,750.2)	(32,765.7)	(12,883.6)	(10,269.5)
Civil servant high	68,482.1	69,003.8$$**$$	117,724.3$$*$$	116,792.8$$***$$	49,398.6$$***$$	46,927.4$$***$$
	(51,736.9)	(23,159.3)	(53,990.3)	(25,315.8)	(11,839.8)	(7934.5)
Fishing	32,699.3	26,684.1	17,591.2	41,879.9	−11,521.8	15,915.0
	(293,053.6)	(147,219.2)	(305,817.4)	(160,927.4)	(67,064.0)	(50,438.1)
Mining and quarrying	96,301.9	−29,378.7	102,114.8	−34,925.6	7973.6	−4619.3
	(85,953.7)	(104,286.2)	(89,697.4)	(113,996.8)	(19,670.1)	(35,729.0)
Manufacturing	−31,429.4	13,513.8	−28,964.0	16,103.5	4458.8	3853.5
	(20,178.1)	(11,141.2)	(21,057.0)	(12,178.6)	(4617.7)	(3817.0)
Electricity, gas and water	−22,665.0	−31,920.7	−36,084.9	−34,396.2	−11,088.7	−1274.6
	(43,521.0)	(30,701.9)	(45,416.6)	(33,560.7)	(9959.6)	(10,518.6)
Construction	−60,415.5$$*$$	26,941.6	−63,211.6$$**$$	32,745.1	−1318.4	6792.5
	(23,479.7)	(18,966.9)	(24,502.3)	(20,733.0)	(5373.2)	(6498.2)
Wholesale and retail	−55,029.1$*$	−8557.9	−56,965.3$*$	−13,231.4	−704.1	−3690.5
	(25,122.9)	(10,611.9)	(26,217.1)	(11,600.0)	(5749.3)	(3635.7)
Hotels and restaurants	−59,293.4	−21,015.1	−55,862.9	−15,556.8	4592.6	6228.2
	(43,284.0)	(16,606.9)	(45,169.2)	(18,153.3)	(9905.3)	(5689.6)
Transp., storage and com.	−34,546.5	−2795.9	−38,894.0	−2754.8	−2439.0	1288.0
	(26,252.7)	(15,337.6)	(27,396.2)	(16,765.8)	(6007.8)	(5254.8)
Financial intermediation	−14,329.6	27,482.8	−7122.6	39,991.2$$*$$	9508.2	13,482.0$$**$$
	(31,536.3)	(14,957.5)	(32,909.8)	(16,350.2)	(7216.9)	(5124.5)
Real estate	8,143.7	11,979.5	6874.9	12,143.2	734.7	1203.6
	(23,465.8)	(11,545.5)	(24,487.8)	(12,620.5)	(5370.0)	(3955.5)
Public adm. and defence	−33,559.1	−16,819.1	−59,131.0	−28,413.7$$*$$	−23,432.4$$***$$	−9894.9$$*$$
	(29,628.4)	(12,872.2)	(30,918.8)	(14,070.8)	(6780.3)	(4410.1)
Education	−44,289.0	−13,541.9	−59,525.8	−12,574.7	−12,505.7	1520.9
	(31,481.7)	(11,351.8)	(32,852.8)	(12,408.8)	(7204.4)	(3889.2)
Health and social work	−48,569.1	6,517.2	−59,362.9$*$	2009.5	−8358.1	−3434.5
	(27,592.1)	(10,044.2)	(28,793.9)	(10,979.4)	(6314.3)	(3441.2)
Other ser. act.	−90,201.7$$**$$	−11,378.9	−98,597.2$$**$$	−18,103.6	−6647.5	−5657.7
	(30,426.5)	(13,617.6)	(31,751.7)	(14,885.6)	(6963.0)	(4665.5)
Activities of households	−127,822.3	978.9	−136,304.6	1268.8	−6376.4	1002.6
	(290,462.7)	(27,055.3)	(303,113.7)	(29,574.5)	(66,471.1)	(9269.3)
Extra-territorial org.	33,404.6	−66,503.6	28,076.6	−76,349.7	−3906.7	−8602.4
	(169,038.5)	(74,080.7)	(176,400.9)	(80,978.7)	(38,683.7)	(25,380.5)
No coworkers	−222,898.1$$***$$	−17,075.5	−225,739.6$$***$$	−19,513.6	−4633.7	−2551.2
	(29,114.2)	(15,422.8)	(30,382.2)	(16,858.8)	(6662.6)	(5283.9)
Small company	−9127.1	24,264.5$$***$$	−11,260.3	19,283.6$$*$$	−1912.2	−5011.7$$*$$
	(15,814.0)	(6931.7)	(16,502.8)	(7577.1)	(3619.0)	(2374.8)
Large company	−26,240.4$$*$$	6355.9	−15,634.4	13,679.2$$*$$	10,687.9$$***$$	6935.6$$**$$
	(12,469.9)	(6207.0)	(13,013.0)	(6784.9)	(2853.7)	(2126.5)
Cohabiting	11,890.3	−23,016.8$$**$$	8854.2	−25,169.3$$**$$	−2861.2	−1887.4
	(16,522.2)	(8031.5)	(17,241.8)	(8779.4)	(3781.0)	(2751.6)
Single	32,121.7	−21,475.1$$**$$	33,272.9	−23,106.7$$**$$	1442.5	−1234.8
	(17,083.6)	(7,818.7)	(17,827.7)	(8546.7)	(3909.5)	(2678.7)
Divorced/separated	−3415.6	−45,187.9$$***$$	−10,456.2	−40,941.2$$***$$	−7416.9$$*$$	4591.6$$*$$
	(15,973.1)	(6382.5)	(16,668.8)	(6976.8)	(3655.4)	(2186.7)
Widowed	−32,453.8	1538.9	−61,455.8	13,813.4	−28,794.5	12,648.9$*$
	(88,012.4)	(14,448.5)	(91,845.7)	(15,793.8)	(20,141.2)	(4950.1)
Exp, full-time	3399.3$$**$$	741.1	7176.5$$***$$	4342.8$$***$$	3872.3$$***$$	3561.9$$***$$
	(1035.8)	(378.8)	(1080.9)	(414.0)	(237.0)	(129.8)
Exp, part-time	−3040.8	1081.7$$*$$	−970.2	2799.0$$***$$	2064.9$$***$$	1712.8$$***$$
	(2183.3)	(464.8)	(2278.4)	(508.1)	(499.6)	(159.3)
Exp, unemployed	−4561.5	−4709.7$$***$$	−3959.7	−4665.6$$***$$	777.2	42.1
	(2363.9)	(869.0)	(2466.9)	(949.9)	(541.0)	(297.7)
Has statutory pensions	23,094.7	−5744.8	21,151.6	−5684.9	−1701.8	324.4
	(19,316.5)	(9779.9)	(20,157.8)	(10,690.6)	(4420.5)	(3350.7)
Has civil servant pension	−50,218.0	−5824.4	12,243.6	38,328.4	62,662.9$$***$$	43,804.6$$***$$
	(40,305.7)	(18,822.6)	(42,061.2)	(20,575.3)	(9223.8)	(6448.7)
Has occupational pension	3816.1	4480.7	47,337.8$$***$$	34,724.5$$***$$	43,157.9$$***$$	30,423.2$$***$$
	(11,321.6)	(5473.2)	(11,814.7)	(5982.9)	(2590.9)	(1875.2)
Educ. flag	−3320.1	17,253.5	11,501.3	16,440.2	15,053.4	−892.2
	(41,675.5)	(19,333.1)	(43,490.7)	(21,133.3)	(9537.3)	(6623.6)
Marst. flag	57,544.0	−97,676.4	62,305.8	−142,312.9	5844.2	−43,691.9
	(129,983.6)	(85,165.7)	(135,645.0)	(93,095.8)	(29,746.2)	(29,178.2)
miss_expft12	−1854.6	−35,072.6	−81,569.9	−25,095.0	−77,514.4	9694.8
	(293,679.9)	(104,414.7)	(306,471.0)	(114,137.3)	(67,207.3)	(35,773.1)
Comp. size flag	−56,890.9$*$	12,298.2	−49,612.4	14,706.5	7080.1	3114.5
	(24,919.5)	(10,451.5)	(26,004.9)	(11,424.6)	(5702.7)	(3580.7)
Occup. flag	82,073.0	44,671.4	166,476.4$$**$$	56,086.0	69,735.5$$***$$	11,711.2
	(60,646.7)	(27,269.3)	(63,288.2)	(29,808.5)	(13,878.7)	(9342.6)
Constant	7372.9	51,299.6$$**$$	−47.4	63,356.9$$**$$	−12,153.7	11,011.2
	(46,769.3)	(18,841.0)	(48,806.3)	(20,595.3)	(10,702.9)	(6455.0)
Observations	4047	4847	4047	4847	4047	4847

OLS regression estimates utilising the SOEPv30 2012 and 2013. The sample includes non-retired individuals between 25 and 60 years old and sample weights. Each column includes the estimates for indicated wealth variable as the dependent variable and the indicated gender. OLS regression estimated using the full set of controls described in Appendix A. Standard errors in parenthesis

$$*$$ $$p<0.05$$, $$**$$ $$p<0.01$$, $$***$$ $$p<0.001$$

**Table 15:** Determinants of accumulated pension wealth, by gender

	Statutory		Occupational		Civil	
	Male	Female	Male	Female	Male	Female
Number of children	922.4	2565.7$$***$$	7769.5	496.7	3659.4	576.8
	(661.5)	(509.7)	(4985.0)	(5357.5)	(2283.0)	(1538.5)
Children in household	−3699.6$*$	−1325.3	−11,277.9	6068.4	3080.7	−4470.6
	(1668.1)	(1224.0)	(11,911.4)	(11,218.1)	(5441.8)	(3400.6)
Immigrant	−9679.5$$***$$	−7541.4$$***$$	−13,806.6	−8180.0	−11,985.9	−5728.8
	(2901.6)	(2002.2)	(51,006.9)	(73,163.5)	(12,115.9)	(8716.9)
East	−10,310.9$$***$$	−5456.4$$***$$	−9342.6	−21,909.6	−19,333.3$$***$$	−4299.1
	(1483.4)	(1208.2)	(12,414.7)	(12,007.4)	(5565.4)	(3453.5)
Age cohort 25-36	−19,110.6$$***$$	-23,880.6$$***$$	−61,940.3$$*$$	-16,720.6	3552.5	1622.5
	(3445.9)	(2015.2)	(26,740.8)	(21,858.3)	(12,396.3)	(6156.7)
Age cohort 37-48	−23,079.2$$***$$	-22,598.5$$***$$	−67,735.6$$***$$	−55,977.4$$***$$	−8216.1	−1518.0
	(2083.2)	(1437.5)	(16,543.6)	(15,137.8)	(7159.9)	(4057.1)
Lower vocational	47.5	−905.4	37,553.1	−27,321.6	168.1	1876.4
	(2570.4)	(1691.9)	(29,110.0)	(75,139.7)	(12,482.8)	(6537.3)
Upper vocational	−499.7	−479.0	59,126.6	−24,709.4	14,059.6	1795.0
	(2952.1)	(1978.6)	(30,902.3)	(75,125.1)	(13,129.2)	(6975.0)
University	18,790.3$$***$$	4524.3$$*$$	47,681.8	−14,394.3	47,395.1$$***$$	13,090.7
	(3000.3)	(1957.5)	(30,853.3)	(74,286.4)	(13,213.8)	(6917.5)
Not employed	−3758.5	−2783.2	72,114.6	−57,256.7	−1745.7	4,402.1
	(3049.9)	(1964.7)	(61,391.3)	(67,924.0)	(18,727.1)	(7856.6)
Trainee	5189.1	2690.0	−17,597.2	−31,402.2	31,132.8	2500.9
	(6216.7)	(4794.1)	(67,207.1)	(98,266.3)	(27,034.9)	(17,884.1)
Self-employed	−21,993.9$$***$$	−10,706.1$$***$$	168,455.5$$***$$	−50,627.2	14,436.6	27,997.6$$*$$
	(2988.6)	(2790.2)	(48,514.2)	(97,344.2)	(13,482.0)	(11,003.3)
White collar	6427.2$$***$$	3328.4$$*$$	38,866.4	−102,588.2	11,524.1	8185.0
	(1,672.1)	(1,527.3)	(35,190.6)	(66,643.4)	(6,086.4)	(5,404.8)
Civil servant low	18,871.6	−7960.9	83,113.1$*$	−33,341.2	22,179.4	16,298.6
	(20,783.0)	(21,541.9)	(34,952.2)	(66,907.4)	(24,487.9)	(15,467.8)
Civil servant high	1924.3	−5525.3	126,263.5$$***$$	−4624.6	−5070.5	20,985.5
	(12,997.4)	(8967.5)	(36,103.6)	(66,351.0)	(17,866.7)	(10,762.0)
Fishing	11,286.7	6568.2			37,221.9	
	(36,352.4)	(30,395.2)			(76,279.1)	
Mining and quarrying	11,100.6	2339.3			−5866.5	−23,081.1
	(10,701.9)	(21,537.3)			(31,411.6)	(47,981.7)
Manufacturing	2834.6	2098.8	176,411.8$$**$$		4,332.4	8,570.5
	(2698.9)	(2447.4)	(53,850.6)		(10,377.6)	(7,181.6)
Electricity, gas and water	−5139.6	5955.2			−7,832.0	−10,284.6
	(5562.2)	(6502.9)			(16,225.1)	(13,608.6)
Construction	2629.9	8153.2$*$	−13,590.2		−15,658.6	−7846.2
	(3102.4)	(4044.8)	(50,369.6)		(13,310.6)	(13,296.1)
Wholesale and retail	−196.3	−4529.9	86,374.6	216,009.1$$***$$	10,065.2	−5802.2
	(3375.2)	(2331.8)	(66,033.2)	(64,105.4)	(14,046.4)	(7698.2)
Hotels and restaurants	1059.4	1000.3	55,239.6	679,686.2$$***$$	27,178.7	−21,984.1
	(5670.7)	(3592.6)	(87,178.2)	(76,258.3)	(29,496.5)	(15,017.1)
Transp., storage and com.	373.0	3911.2	−21,927.6	−14,124.2	−5700.1	571.3
	(3482.1)	(3,420.5)	(34,542.5)	(34,594.8)	(12,958.0)	(9,226.1)
Financial intermediation	7534.8	3385.2	6543.1	49,788.4	−4717.4	13,012.7
	(4097.3)	(3216.3)	(58,185.6)	(76,832.3)	(12,478.9)	(7520.6)
Real estate	318.5	1587.9	−11,250.8	−6215.2	6235.0	1051.8
	(3186.5)	(2552.7)	(38,757.4)	(41,810.0)	(12,007.0)	(8348.0)
Public adm. and defence	−1893.1	875.0	−31,052.2	818.3	−24,828.5	−12,367.5
	(4363.7)	(2964.8)	(28,151.2)	(18,754.0)	(12,802.4)	(6965.1)
Education	−4,768.9	−488.3	−14,885.7	34,316.4	−21,890.3	−9,053.8
	(4653.2)	(2592.8)	(29,926.9)	(17,875.4)	(14,116.2)	(6751.8)
Health and social work	81.8	−2093.8	45,157.2	−8297.8	−7451.7	−5862.5
	(3687.0)	(2230.6)	(55,772.2)	(30,937.5)	(12,367.1)	(6401.5)
Other ser. act.	−2214.2	−3944.0	−86,375.1	−1786.3	−12,887.4	−5972.6
	(4065.1)	(3003.7)	(44,979.1)	(40,503.9)	(13,906.8)	(8449.0)
Activities of households	−10,147.4	−1991.3				−25,850.1
	(36,008.2)	(5,705.2)				(48,061.2)
Extra-territorial org.	619.6	−9732.2	57,107.2		−39,576.8	−261.2
	(20,904.2)	(15,313.6)	(101,130.0)		(52,872.7)	(34,490.4)
No coworkers	−298.3	−544.2	−106,927.9	−15,315.9	−3279.7	9914.5
	(4165.0)	(3627.2)	(86,547.7)	(73,479.3)	(21,466.9)	(15,544.0)
Small company	−2570.0	−,236.5$$***$$	−19,634.4	19,198.6	10,135.4	−7297.4
	(2036.4)	(1488.8)	(31,356.5)	(19,154.0)	(9099.2)	(4701.4)
Large company	7793.5$$***$$	5892.4$$***$$	−26,321.8$$*$$	−4181.5	13,098.8$$*$$	2478.6
	(1620.8)	(1359.6)	(13,095.1)	(10,717.1)	(5449.4)	(3305.4)
Cohabiting	−3217.3	−208.7	−20,805.9	−8232.5	7105.6	−3812.3
	(2198.0)	(1795.6)	(18,413.0)	(15,533.7)	(7537.6)	(4662.2)
Single	−563.2	−1475.8	−11,814.0	−4793.6	−248.2	−3710.6
	(2283.1)	(1771.5)	(19,677.9)	(14,120.1)	(8222.2)	(4899.2)
Divorced/separated	−4256.6$*$	3489.9$*$	−18,607.4	−30,113.8$*$	−1686.4	6169.8
	(2106.0)	(1368.3)	(15,090.5)	(13,906.6)	(7188.6)	(3854.1)
Widowed	−7773.8	6001.9$$*$$		110,036.2$$**$$	801.6	19,434.6$$*$$
	(11,437.5)	(3054.3)		(41,671.0)	(42,638.1)	(9314.0)
Exp, full-time	2926.4$$***$$	2790.9$$***$$	6772.2$$***$$	10,088.2$$***$$	2633.5$$***$$	1299.0$$***$$
	(137.5)	(81.3)	(1,098.4)	(859.9)	(486.4)	(249.0)
Exp, part-time	1494.9$$***$$	1365.9$$***$$	7827.0$$**$$	4675.3$$***$$	453.5	986.6$$**$$
	(288.8)	(99.2)	(2658.1)	(1040.4)	(995.3)	(302.9)
Exp, unemployed	266.1	103.4	−6602.0	847.5	824.2	−424.5
	(304.5)	(183.0)	(3510.6)	(7976.5)	(2153.6)	(1300.8)
Educ. flag	3768.5	360.2	167,610.6$$**$$	−14,368.0	17,114.7	1359.0
	(6267.5)	(4561.6)	(55,013.1)	(90,561.2)	(29,044.3)	(16,545.1)
Marst. flag	−6006.8	−13,408.6			38,702.1	
	(17,993.0)	(21,461.7)			(42,561.8)	
Comp. size flag	7270.4$*$	1523.3	34,269.3	9010.4	−10,272.2	−1145.2
	(3384.2)	(2343.0)	(36,872.7)	(17,882.5)	(14,327.9)	(7102.2)
Occup. flag	46,604.0$$***$$	11,800.3$$*$$	−125,883.8		31,135.9	299.2
	(7683.9)	(5817.4)	(87,917.4)		(22,371.4)	(15,395.7)
miss_expft12		2125.2	−116,816.8			
		(30,644.8)	(97,317.2)			
Constant	8134.8	19,823.4$$***$$	−71,676.9	23,282.1	−47,135.6$$*$$	−5030.7
	(6020.9)	(3717.9)	(60,176.2)	(102,755.6)	(23,587.9)	(12,636.4)
Observations	3427	4261	336	332	1259	1413

Estimated using the SOEPv30 2012 and 2013 sample including non-retired individuals between 25 and 60 years old. Each column includes the estimates for indicated wealth variable as the dependent variable. Decomposition estimated using the full set of controls described in Appendix A

$$*$$ $$p<0.05$$, $$**$$ $$p<0.01$$, $$***$$ $$p<0.001$$

**Table 16:** Oaxaca–Blinder decomposition at means of the gender wealth gap, pension wealth and augmented Wealth

	(1)	(2)	(3)
	Net wealth	Augmented wealth	Pension wealth
Male	114,118.9$$***$$	194,278.4$$***$$	79,994.0$$***$$
Female	72,699.3$$***$$	135,788.2$$***$$	62,932.0$$***$$
Difference	41,419.6$$***$$	58,490.2$$***$$	17,062.0$$***$$
Explained	9,272.1$$*$$	27,391.1$$***$$	18,248.5$$***$$
Unexplained	32,147.5$$***$$	31,099.1$$***$$	−1186.5
Explained			
Number of children	−1,717.5$$*$$	−2476.9$$**$$	−758.4$$***$$
Children in household	−265.7	−171.4	87.6
Immigrant	475.7$*$	561.3$*$	84.0$*$
East	−548.3	−667.9	−117.2
East/female interaction	−4644.8$$*$$	−6365.6$$**$$	−1631.3$$*$$
Age cohort 25–36	306.7	432.0	124.3
Age cohort 37–48	157.9	301.0	143.2
Lower vocational	38.9	28.9	−10.2
Upper vocational	67.6	56.3	−11.6
University	1260.9	1538.7$*$	269.0
Not employed	−4,163.8$$***$$	−4531.1$$***$$	−428.6$$*$$
Trainee	165.7	240.8	77.7
Self-employed	11,907.6$$***$$	10,811.7$$***$$	−1018.2$$***$$
White collar	−3593.6$$***$$	−4827.7$$***$$	−1245.5$$***$$
Civil servant low	542.4$*$	629.8$*$	92.0
Civil servant high	305.8	526.8	220.3
Industry	2014.8	3265.8	1431.5$*$
Company	−3,171.2$$***$$	−2,166.3$$*$$	967.2$$***$$
Cohabiting	−133.8	−180.0	−42.5
Single	−153.8	−185.0	−16.4
Divorced/separated	1215.1$$***$$	1215.9$$**$$	−0.2
Widowed	32.4	−126.8	−164.1
Exp, full-time	12,106.5$$***$$	40,049.7$$***$$	27,941.2$$***$$
Exp, part-time	−5807.9$$*$$	−14,265.6$$***$$	−8510.7$$***$$
Exp, unemployed	1027.9$$**$$	1002.0$$**$$	−32.5
Has statutory pensions	−186.7	−198.1	−21.3
Has civil servant pension	−343.1	386.4	726.8$*$
Has occupational pension	95.7	804.5	706.7$*$
Educ. flag	12.1	18.5	6.4
Marst. flag	−19.7	−27.2	−7.3
Exp. FT flag	5.9	8.5	2.5
Exp. PT flag	0.0	0.0	0.0
Exp. UE flag	0.0	0.0	0.0
Comp. size flag	2,313.3	1,755.2	−596.4
Occup. flag	−30.9	−53.1	−19.4
Unexplained			
Number of children	11,818.1	12,056.7	−183.5
Children in household	1016.4	290.1	−558.6
Immigrant	−292.3	−521.6	−226.1
East	−6234.1$$**$$	−7636.8$$**$$	−1352.0$$*$$
East/female interaction	4644.8$$*$$	6365.6$$**$$	1631.3$$*$$
Age cohort 25 −36	11,684.5$*$	12,226.7$*$	1121.1
Age cohort 37 −48	8630.7	7050.4	−1413.4
Lower vocational	1366.4	2381.2	857.0
Upper vocational	651.7	1280.9	612.6
University	13,891.5$$**$$	20,331.3$$***$$	6370.3$$***$$
Not employed	−430.1	−201.7	376.0
Trainee	183.8	356.0	203.7
Self-employed	19,464.3$$***$$	18,528.3$$***$$	−760.7
White Collar	−1055.9	3625.7	4729.4$$**$$
Civil servant low	819.1	731.2	−100.9
Civil servant high	−39.2	14.1	100.6
Industry	−32,129.3	−35,309.7	−2520.3
Company	−27,014.4$$**$$	−25,250.2$$*$$	1,937.7
Cohabiting	3870.5$*$	3779.2$*$	−101.3
Single	8962.7$$**$$	9425.3$$**$$	448.3
Divorced/separated	4,951.4$$*$$	3596.2	−1443.6$$**$$
Widowed	−155.5	−353.7	−201.2$*$
Exp, full-time	44,990.9$$**$$	48,948.2$$**$$	6244.5
Exp, part-time	−3335.9	−2895.3	514.3
Exp, unemployed	−52.8	534.7	775.6
Has statutory pensions	24,793.6	23,106.9	−1705.0
Has civil servant pension	−3427.3	−1995.2	1475.4
Has occupational pension	−214.8	3799.1	3850.5$$***$$
Educ. flag	−309.4	−73.5	240.6$*$
Marst. flag	151.2	192.3	41.5
Exp. FT flag	8.1	−18.3	−25.6
Exp. PT flag	0.0	0.0	0.0
Exp. UE flag	0.0	0.0	0.0
Comp. size flag	−11,366.6$$**$$	−10,542.3$$*$$	682.3
Occup. flag	231.9	681.7	358.0
Observations	8894	8894	8894
Omitted high ed, high civil servant, married			

Estimated using the SOEPv30 2012 and 2013 sample including non-retired individuals between 25 and 60 years old. Each column includes the estimates for indicated wealth variable as the dependent variable. Decomposition estimated using the full set of controls described in Appendix A

$$*$$ $$p<0.05$$, $$**$$ $$p<0.01$$, $$***$$ $$p<0.001$$

**Table 17:** RIF-OAXACA decomposition of net wealth gender gap, population 25-60

	(1)	(2)	(3)
	Q25	Q50	Q90
Male	1,755.92$$*$$	32,985.83$$***$$	261,051.92$$***$$
Female	171.11	20,514.79$$***$$	191,978.74$$***$$
Difference	1584.81	12,471.04$$***$$	69,073.18$$***$$
Explained	9,974.06$$***$$	25,674.91$$***$$	117,993.01$$***$$
unexplained	−8389.25$$***$$	−13,203.87$$***$$	−48,919.83$$**$$
Explained			
Number of children	86.76	−178.07	−2664.54
Children in household	8.31	−185.84	−1,433.23
Immigrant	166.82$*$	260.88$*$	1188.68
East	−1.31	−111.94	−1150.77
East/female interaction	0.00	0.00	0.00
Age cohort 25-36	38.81	68.91	750.16
Age cohort 37-48	−4.90	−13.45	540.23
Lower vocational	22.94	15.95	21.93
Upper vocational	40.70	48.00	156.02
University	408.86	791.84	3681.91
Not employed	1015.74$$*$$	−148.33	−8170.27$$**$$
Trainee	−1.89	1.10	542.79
Self-employed	984.34$$***$$	2716.13$$***$$	27,333.87$$***$$
White Collar	−788.33$$**$$	−2555.26$$***$$	−5355.19$$*$$
Civil servant low	85.59	465.04$*$	1356.17
Civil servant high	7.14	191.46	524.40
Industry	1529.25$$**$$	3189.71$$**$$	9490.93
Company	−41.05	9.22	−6308.99$*$
Cohabiting	5.06	−228.52	348.94
Single	−215.13	−425.13	2,944.93$*$
Divorced/separated	592.95$$***$$	1126.31$$***$$	198.32
Widowed	14.56	−274.47	4818.46$$***$$
Exp, full-time	4187.19$$***$$	18,466.94$$***$$	24,392.28
Exp, part-time	311.00	1258.51	59,949.13$$***$$
Exp, unemployed	664.25$$**$$	610.70$$**$$	1456.04$$*$$
Has statutory pensions	−322.87$$**$$	−180.52	−1455.41
Has civil servant pension	241.72$*$	−48.36	−391.70
Has occupational pension	42.14	99.86	572.17
Educ. flag	−0.41	−2.42	−16.75
Marst. flag	9.11	14.10	298.28
Exp. FT flag	−7.39	8.89	4.21
Exp. PT flag	0.00	0.00	0.00
Exp. UE flag	0.00	0.00	0.00
Comp. size flag	894.58$*$	693.98	4,507.47
Occup. Flag	−0.50	−10.30	−137.47
Unexplained			
Number of children	108.10	−339.04	30,899.19
Children in household	406.66	2056.94	1778.41
Immigrant	−23.62	−90.93	−1303.23
East	439.36	−910.90	−7,300.27
East/female interaction	0.00	0.00	0.00
Age cohort 25-36	352.38	3372.27	7254.82
Age cohort 37-48	965.72	3,401.99$*$	−364.74
Lower vocational	−2,397.08	−467.35	−5,927.41
Upper vocational	−1,109.75	−298.88	1,981.38
University	−296.95	4804.39$$**$$	22,003.17$$*$$
Not employed	−2403.66$$**$$	−1525.75	−818.45
Trainee	−205.24	−158.38	1248.01
Self-employed	557.88$$*$$	1983.16$$***$$	23,987.16$$***$$
White collar	−848.64	5,908.14$*$	−8,145.85
Civil servant low	−35.05	235.20	892.65
Civil servant high	−549.95	254.41	−233.03
Industry	−6636.91$$*$$	−12,254.15$$**$$	−38,872.49
Company	−2,443.88	−2,543.95	−14,248.07
Cohabiting	766.41$*$	199.12	6723.08$*$
Single	916.55$$*$$	1,448.30$$*$$	12,664.38$$**$$
Divorced/Separated	168.17	−128.81	12,088.41$*$
Widowed	155.71	408.20	−5,508.47$$***$$
Exp, full-time	5,110.29$$**$$	22,309.74$$***$$	25,566.01
Exp, part-time	−1500.13	−4331.60	−82,330.98$$***$$
Exp, unemployed	−1622.00$$**$$	−831.65	275.14
Has statutory pensions	7704.91$*$	−840.11	70,593.38
Has civil servant pension	1089.10$*$	293.50	−1574.09
Has occupational pension	−946.01	−418.14	10,051.61
Educ. flag	−211.79	−208.88	−395.73
Marst. flag	18.33	37.17	460.67
Exp. FT flag	43.62	−7.90	11.02
Exp. PT flag	0.00	0.00	0.00
Exp. UE flag	0.00	0.00	0.00
Comp. size flag	−2,149.27$*$	−2,105.68	−17,091.93
Occup. flag	−66.78	−8.82	947.52
Observations	8894	8894	8894

Estimated using the SOEPv30 2012 and 2013 sample including non-retired individuals ages between 25 and 60 and sample weights. Decomposition estimated using the full set of controls described in Appendix A

$$*$$ $$p<0.05$$, $$**$$ $$p<0.01$$, $$***$$ $$p<0.001$$

**Table 18:** RIF-OAXACA decomposition of augmented wealth gender gap, population 25-60

	(1)	(2)	(3)
	Q25	Q50	Q90
Male	35,606.59$$***$$	104,686.24$$***$$	441,057.15$$***$$
Female	27,941.02$$***$$	82,124.06$$***$$	308,558.83$$***$$
Difference	7665.57$$***$$	22,562.18$$***$$	132,498.33$$***$$
Explained	23,249.64$$***$$	58,872.41$$***$$	163,094.04$$***$$
Unexplained	−15,584.07$$***$$	−36,310.23$$***$$	−30,595.72
Explained			
Number of children	−22.48	641.83	−7095.15$*$
Children in household	−86.24	−383.45	−485.24
Immigrant	244.90$*$	647.60$*$	1222.06
East	−78.84	−221.59	−1458.97
East/female interaction	0.00	0.00	0.00
Age cohort 25-36	222.70	294.90	495.59
Age cohort 37-48	6.03	133.90	681.82
Lower vocational	47.95	−16.87	50.00
Upper vocational	77.38	1.75	273.26
University	812.71	1,101.35	5,833.68
Not employed	1,499.95$*$	900.11	−8048.31$*$
Trainee	−83.33	147.74	829.64
Self-employed	880.63$$**$$	3846.35$$***$$	28,278.19$$***$$
White collar	−698.28	−3837.45$$***$$	−10,085.18$$***$$
Civil servant low	451.99$*$	757.50$*$	315.13
Civil servant high	94.16	301.55	1200.32
Industry	2054.46$$*$$	8049.85$$***$$	−920.16
Company	130.10	317.84	−5415.89
Cohabiting	−79.55	−312.94	490.93
Single	−891.50$$**$$	−325.55	3515.83$$*$$
Divorced/separated	144.87	1192.70$$**$$	498.09
Widowed	−349.81	−347.92	6138.13$$***$$
Exp, full-time	23,926.88$$***$$	54,712.18$$***$$	93,072.09$$***$$
Exp, part-time	−4340.78	−11,131.69$*$	42,459.51$*$
Exp, unemployed	512.20$$*$$	928.45$$**$$	73.07
Has statutory pensions	−1094.18$$***$$	25.30	178.73
Has civil servant pension	370.28	395.38	2585.22
Has occupational pension	286.40	628.29	2691.74
Educ. flag	−0.26	5.72	114.86
Marst. flag	11.50	21.04	73.62
Exp. FT flag	10.65	13.95	67.67
Exp. PT flag	0.00	0.00	0.00
Exp. UE flag	0.00	0.00	0.00
Comp. size flag	−817.29	410.30	5,708.07
Occup. flag	6.45	−25.71	−244.30
Unexplained			
Number of children	−4803.58	−12,930.50$$**$$	50,077.77$$*$$
Children in household	4,918.82$$**$$	5819.44$$*$$	−15,175.59
Immigrant	638.57	−474.11	−2332.24
East	1699.05	−718.40	−14,451.73$*$
East/female interaction	0.00	0.00	0.00
Age cohort 25-36	2081.30	8702.96$*$	19,077.75
Age cohort 37-48	1532.04	6591.51$*$	7448.75
Lower vocational	−2311.82	−9354.84$*$	19,524.06
Upper vocational	−1427.58	−4348.91$*$	15,641.70$*$
University	945.75	6562.39$$*$$	58,280.20$$***$$
Not employed	−1389.03	−5,346.70$*$	−257.88
Trainee	−349.10	−19.33	1866.50
Self-employed	804.62	3312.24$$***$$	26,155.26$$***$$
White collar	2365.78	6702.93	4706.49
Civil servant low	106.94	99.86	228.04
Civil servant high	−476.43	−309.19	1365.57
Industry	−7715.14	−19,260.07$*$	−72,836.79
Company	−983.45	−6160.92	−10,706.74
Cohabiting	802.95	519.30	7858.98$*$
Single	1,092.74	2111.38	13,729.15$$**$$
Divorced/separated	942.54	−698.67	8686.25
Widowed	362.44	191.91	−7575.11$$***$$
Exp, full-time	13,957.32$$***$$	39,315.01$$***$$	46,580.09
Exp, part-time	−2177.83	−2138.58	−73,618.22$$**$$
Exp, Unemployed	−2099.61$*$	−25.58	7506.50
Has statutory pensions	−6,976.10	−10,499.06	64,841.92
Has civil servant pension	345.96	1,603.27	7235.44
Has occupational pension	−1243.69	61.90	28,045.80$$**$$
Educ. flag	−397.04$*$	−386.44	1,722.93$*$
Marst. flag	44.92	41.11	309.65
Exp. FT flag	7.17	−15.27	−181.11
Exp. PT flag	0.00	0.00	0.00
Exp. UE flag	0.00	0.00	0.00
Comp. size flag	540.38	−3,596.49	−21,638.30
Occup. flag	−73.03	147.53	2447.53
Observations	8894	8894	8894

Estimated using the SOEPv30 2012 and 2013 sample including non-retired individuals ages between 25 and 60 and sample weights. Decomposition estimated using the full set of controls described in Appendix A

$$*$$ $$p<0.05$$, $$**$$ $$p<0.01$$, $$***$$ $$p<0.001$$

**Table 19:** RIF-OAXACA decomposition of pension wealth gender gap, population 25-60

	(1)	(2)	(3)
	Q25	Q50	Q90
Male	18,591.39$$***$$	52,141.44$$***$$	180,664.76$$***$$
Female	16,701.36$$***$$	41,046.49$$***$$	143,067.56$$***$$
Difference	1,890.02$$*$$	11,094.95$$***$$	37,597.20$$***$$
Explained	7,942.37$$***$$	19,013.44$$***$$	46,576.87$$***$$
unexplained	−6052.35$$***$$	−7918.48$$**$$	−8979.67
Explained			
Number of children	−15.73	−35.50	−1790.61
Children in household	−16.11	−51.87	−7.81
Immigrant	57.28	185.77$*$	110.37
East	−36.29	−74.25	−282.92
East/female interaction	0.00	0.00	0.00
Age cohort 25-36	118.39	225.89	−22.03
Age cohort 37-48	−7.29	139.92	352.58
Lower vocational	15.00	−9.83	9.23
Upper vocational	25.03	−10.56	−16.92
University	203.05	184.13	1187.96
Not employed	570.61	623.56	−1524.12
Trainee	19.07	60.52	216.55
Self-employed	−957.88$$***$$	−1418.98$$***$$	−1068.14
White collar	−68.65	−686.15$$*$$	−3632.68$$**$$
Civil servant low	374.53$$**$$	320.36$$*$$	403.40
Civil servant high	126.93	124.10	837.35
Industry	1,004.45$$*$$	2541.05$$***$$	−1261.64
Company	347.82$$*$$	634.19$$**$$	2,308.72$$*$$
Cohabiting	8.29	−77.96	38.10
Single	−285.16$*$	41.09	853.43
Divorced/separated	−163.44$$**$$	−52.22	793.24
Widowed	155.22	−72.10	2498.61$$***$$
Exp, full-time	10,992.36$$***$$	23,626.76$$***$$	64,286.71$$***$$
Exp, part-time	−3361.49$$*$$	−7058.07$$**$$	−20,813.97$$*$$
Exp, unemployed	−47.63	119.57	−490.76
Has statutory pensions	−1383.80$$***$$	−321.99$$**$$	1768.02$$***$$
Has civil servant pension	311.24$*$	266.12	1517.82
Has occupational pension	231.33$*$	517.26$*$	1897.14$*$
Educ. flag	−5.48	9.73	33.83
Marst. flag	−3.98	−13.49	13.77
Exp. FT flag	−12.73	5.60	47.51
Exp. PT flag	0.00	0.00	0.00
Exp. UE flag	0.00	0.00	0.00
Comp. size flag	−256.56	−727.15	−1,558.09
Occup. flag	3.99	−2.08	−127.80
Unexplained			
Number of children	−809.58	−3713.71$*$	−558.26
Children in household	1452.96$*$	2953.97$*$	871.34
Immigrant	94.35	−201.44	−647.26
East	320.99	−926.51	−5,102.98$*$
East/female interaction	0.00	0.00	0.00
Age cohort 25-36	253.66	−3410.92$*$	5838.62
Age cohort 37-48	1448.00$$*$$	−3615.83$$**$$	−5841.13
Lower vocational	173.76	−4609.75$*$	12,425.10
Upper vocational	−14.06	−1582.14$*$	3495.40
University	549.14	422.66	19,285.55$$***$$
Not employed	−144.41	−743.13	1,984.67
Trainee	113.26	63.71	352.76
Self-employed	−642.89$$**$$	−1165.81$$**$$	747.59
White collar	723.77	1537.11	10,877.06
Civil servant low	86.58	117.11	439.14
Civil servant high	93.66	131.53	5952.94$*$
Industry	−3070.90	−3279.88	−6998.60
Company	−36.71	−2455.07	11,153.47
Cohabiting	134.20	−579.92	609.68
Single	−281.10	95.10	1906.80
Divorced/separated	342.56	−560.38	−4681.36$*$
Widowed	−279.28	−110.24	−2,589.71$$**$$
Exp, full-time	,783.77$$***$$	6530.25$$**$$	4615.59
Exp, part-time	43.01	−3,012.77	8,899.83
Exp, unemployed	−51.80	−1,723.52$$**$$	4,507.00$$*$$
Has statutory pensions	3645.65	−1750.00	−17,020.78
Has civil servant pension	382.08	−92.02	1276.29
Has occupational pension	307.71	1,301.11	7195.99$*$
Educ. flag	−150.66	47.48	576.12
Marst. flag	4.13	−2.58	117.26
Exp. FT flag	44.64	−11.78	−139.52
Exp. PT flag	0.00	0.00	0.00
Exp. UE flag	0.00	0.00	0.00
Comp. size flag	−154.10	602.76	549.35
Occup. flag	−13.99	88.01	1180.05$*$
Observations	8894	8894	8894

Estimated using the SOEPv30 2012 and 2013 sample including non-retired individuals between 25 and 60 years old. Each column includes the estimates for indicated wealth variable as the dependent variable. Decomposition estimated using the full set of controls described in Appendix A

$$*$$ $$p<0.05$$, $$**$$ $$p<0.01$$, $$***$$ $$p<0.001$$

**Table 20:** Oaxaca–Blinder decomposition at means of the statutory pension wealth gap, civil pension wealth, and occupational pension wealth

	Statutory	Occupational	Civil
overall			
Male	53,253.9$$***$$	12,177.6$$***$$	14,562.5$$***$$
Female	46,268.0$$***$$	8190.0$$***$$	8474.0$$***$$
Difference	6985.8$$***$$	3987.6$$***$$	6088.6$$***$$
Explained	11,126.8$$***$$	4002.3$$***$$	3119.4$$***$$
Unexplained	−4141.0$$***$$	−14.7	2,969.1$$**$$
Explained			
Number of children	−407.4$$***$$	−109.9	−241.1$$*$$
Children in household	123.5$$**$$	−2.1	−33.8
Immigrant	82.6$*$	−8.8	10.1
East	−72.5	−7.5	−37.2
East/female interaction	−942.5$*$	−32.3	−656.5
Age cohort 25–36	89.5	22.5	12.3
Age cohort 37–48	97.5	24.3	21.4
Lower vocational	−1.1	−4.7	−4.4
Upper vocational	−2.7	−4.9	−4.0
University	158.9	−31.0	141.1
Not employed	414.9$$**$$	−397.9$$***$$	−445.5$$***$$
Trainee	39.2	4.6	34.0
Self-employed	−924.8$$***$$	−190.0$$*$$	96.6
White collar	−557.7$$***$$	−194.0$$*$$	−493.7$$***$$
Civil servant low	−284.7$$***$$	309.5	67.3
Civil servant high	−136.4	350.2	6.5
Industry	1287.8$$***$$	−710.4$$**$$	854.1$$*$$
Company	668.6$$***$$	−63.2	361.8$$***$$
Cohabiting	−31.8	−7.6	−3.1
Single	55.3	−105.1	33.4
Divorced/separated	−35.4	32.1	3.1
Widowed	−91.3	−28.3	−44.5
Exp, full-time	18,646.6$$***$$	5498.0$$***$$	3796.6$$***$$
Exp, part-time	−5846.3$$***$$	−1709.2$$***$$	−955.2$$**$$
Exp, unemployed	3.7	−11.8	−24.4
Has statutory pensions	−744.0$$***$$	644.5$$***$$	78.2
Has civil servant pension	−107.9	860.0$*$	−25.3
Has occupational pension	141.7	−84.2	649.1$*$
Educ. flag	2.6	1.5	2.3
Marst. flag	−9.0	−7.0	8.8
Exp. FT flag	−3.7	6.5	−0.4
Exp. PT flag	0.0	0.0	0.0
Exp. UE flag	0.0	0.0	0.0
Comp. size flag	−472.2$*$	−43.0	−81.2
Occup. flag	−13.9	1.6	−7.0
Unexplained			
Number of children	−1359.0	−511.4	1686.9
Children in household	−505.7	−1329.5$*$	1276.6
Immigrant	−61.4	−29.5	−135.2
East	−956.8$*$	346.4	−741.6
East/female interaction	942.5$*$	32.3	656.5
Age cohort 25–36	967.0	−263.7	417.8
Age cohort 37–48	−144.7	57.5	−1,326.2
Lower vocational	463.2	384.2	9.7
Upper vocational	81.2	84.0	447.4
University	3,101.5$$***$$	−115.9	3384.7$$***$$
Not employed	88.9	−144.4	431.5$*$
Trainee	102.0	−101.6	203.4$$***$$
Self-employed	−858.8$$**$$	256.3	−158.2
White collar	2,087.9	572.5	2069.0$*$
Civil servant low	−11.6	−161.5	72.3
Civil servant high	−534.7	663.1	−27.7
Industry	−1801.5	1555.5	−2274.3
Company	1208.3	−1597.7	2327.0$*$
Cohabiting	−332.8	−163.6	395.0
Single	−215.4	64.1	599.6
Divorced/separated	−850.3$$**$$	−183.9	−409.5
Widowed	−74.2	−41.6	−85.5$*$
Exp, full-time	1605.0	−2651.0	7290.5$*$
Exp, part-time	423.8	−122.2	212.7
Exp, unemployed	−184.0	60.3	899.3$$***$$
Has statutory pensions	2911.2	−3305.9	−1,310.2
Has civil servant pension	−159.3	1815.5	−180.9
Has occupational pension	−765.3	241.8	4374.1$$***$$
Educ. flag	68.8	76.1	95.6$*$
Marst. flag	−0.7	11.3	30.9
Exp. FT flag	16.9	−43.1	0.5
Exp. PT flag	0.0	0.0	0.0
Exp. UE flag	0.0	0.0	0.0
Comp. Size flag	704.0	40.6	−62.3
Occup. flag	225.6$$**$$	−22.0	154.4
Observations	8894	8894	8894
Omitted high ed, high civil servant, married			

Estimated using the SOEPv30 2012 and 2013 sample including non-retired individuals between 25 and 60 years old. Each column includes the estimates for indicated wealth variable as the dependent variable. Decomposition estimated using the full set of controls described in Appendix A

$$*$$ $$p<0.05$$, $$**$$ $$p<0.01$$, $$***$$ $$p<0.001$$

**Table 21:** RIF-OAXACA decomposition of statutory pension wealth gap

	Statutory Q25	Statutory Q50	Statutory Q90
Gap (%)	7.4%	16.5%	14.3%
Male	10,649.6$$***$$	38,144.4$$***$$	128,505.5$$***$$
Female	11,440.5$$***$$	31,846.0$$***$$	110,066.9$$***$$
Difference	−790.9	6298.5$$***$$	18,438.6$$***$$
Explained	2548.2$$*$$	14,344.3$$***$$	28,070.7$$***$$
Unexplained	−3339.1$$**$$	−8045.8$$***$$	−9632.0
Explained			
Number of children	−27.5	−38.7	−746.0
Children in household	−22.8	−21.0	537.8$*$
Immigrant	17.1	85.5	185.3
East	−26.6	−46.3	−143.8
East/female interaction	0.0	0.0	0.0
Age cohort 25–36	52.7	198.2	−92.1
Age cohort 37–48	−6.8	88.4	175.2
Lower vocational	4.5	4.3	6.5
Upper vocational	15.2	6.7	11.0
University	99.1	183.4	803.8
Not employed	154.8	560.5	52.1
Trainee	10.3	54.0	129.6
Self-employed	−558.7$$***$$	−1341.6$$***$$	−1701.8$$***$$
White collar	71.9	−545.4$$*$$	−2645.1$$***$$
Civil servant low	−190.6$$**$$	−285.9$$**$$	−283.4
Civil servant high	−86.3	−155.9	−223.5
Industry	737.0$*$	960.8	3239.1
Company	242.5$$**$$	757.0$$***$$	2,315.5$$***$$
Cohabiting	−5.7	−12.2	−98.1
Single	−192.6$*$	242.7	71.6
Divorced/separated	−62.0	−92.1	332.2
Widowed	−17.3	−156.5	1,301.6
Exp, full-time	4876.3$$***$$	18,562.2$$***$$	48,170.3$$***$$
Exp, part-time	−683.4	−2,675.9	−21,591.5$$***$$
Exp, unemployed	1.0	134.3	−107.0
Has statutory pensions	−1813.3$$***$$	−926.9$$***$$	107.2
Has civil servant pension	−46.3	−320.7$*$	−334.1
Has occupational pension	11.5	81.0	212.0
Educ. flag	−15.8	9.5	23.4
Marst. flag	−1.0	5.2	−62.6
Exp. FT flag	−6.6	−13.3	−16.1
Exp. PT flag	0.0	0.0	0.0
Exp. UE flag	0.0	0.0	0.0
Comp. Size flag	15.2	−956.0$*$	−1,452.6
Occup. flag	2.4	−1.2	−106.0
Unexplained			
Number of children	−400.1	−3505.6$*$	−1999.8
Children in household	1702.1$$***$$	2901.5$$**$$	−6951.8$$*$$
Immigrant	114.9	367.5	−926.6
East	145.6	46.3	−2,042.2
East/female interaction	0.0	0.0	0.0
Age cohort 25–36	2771.4$$***$$	−2049.3	6,236.3$$*$$
Age cohort 37–48	761.3	−1737.9	2368.4
Lower vocational	−845.1	−2233.5	4685.2
Upper vocational	−106.1	−575.2	2187.8
University	−1.1	564.6	10,287.8$$***$$
Not employed	642.5	24.4	1,232.2
Trainee	132.1	102.4	300.9
Self-employed	−386.3$$*$$	−1012.9$$**$$	−124.0
White collar	18.7	1483.4	10,056.0$*$
Civil servant low	13.8	64.0	436.2
Civil servant high	7.1	−98.1	682.3
Industry	−196.4	−2,564.9	−3403.6
Company	537.7	1,073.6	10,450.2$*$
Cohabiting	−74.1	92.9	−433.0
Single	−404.2	570.2	−580.0
Divorced/separated	88.5	−118.2	−2803.6
Widowed	−18.8	94.1	−1821.8
Exp, full-time	2910.3$$**$$	,810.2$$***$$	−6773.4
Exp, part-time	−1078.8	−4534.8$*$	12,081.7
Exp, unemployed	−209.6	−1607.2$$**$$	1851.2
Has statutory pensions	8230.6$$***$$	7064.4$$*$$	−913.2
Has civil servant pension	−250.1	−1131.7$*$	−2005.5
Has occupational pension	−174.6	−728.5	−1937.1
Educ. flag	−202.6$*$	63.6	207.1
Marst. flag	4.4	15.2	−67.3
Exp. FT flag	28.5	36.4	23.4
Exp. PT flag	0.0	0.0	0.0
Exp. UE flag	0.0	0.0	0.0
Comp. size flag	−30.5	1303.7	2325.0
Occup. flag	−26.5	−10.4	1073.2$$**$$
Observations	8894		

Estimated using the SOEPv30 2012 and 2013 sample including nonretired individuals between 25 and 60 years old. Each column includes the estimates for indicated wealth variable as the dependent variable. Decomposition estimated using the full set of controls described in Appendix A.

$$*$$ $$p<0.05$$, $$**$$ $$p<0.01$$, $$***$$ $$p<0.001$$

**Table 22:** RIF-OAXACA decomposition of gender gap, population 25-60 excluding self-employed

	(1)	(2)	(3)
	Q25	Q50	Q90
*Net wealth*			
Gap(%)	42.4%	30.2%	16.5%
Male	3906.44$$***$$	35,900.00$$***$$	222,585.64$$***$$
Female	2247.44$$**$$	25,037.41$$***$$	185,805.23$$***$$
Difference	1659.00	10,862.59$$***$$	36,780.41$$***$$
Explained	8547.47$$***$$	20,194.23$$***$$	78,481.46$$***$$
Unexplained	−6888.47$$**$$	−9331.63$$*$$	−41,701.05$$**$$
*Augmented wealth*			
Gap(%)	12.7%	15.3%	23.9%
Male	43,429.01$$***$$	113,628.38$$***$$	415,665.01$$***$$
Female	37,903.74$$***$$	96,143.40$$***$$	316,214.74$$***$$
Difference	5525.27$$*$$	17,484.98$$***$$	99,450.28$$***$$
Explained	18,436.02$$***$$	45,143.32$$***$$	122,621.53$$***$$
Unexplained	−12,910.76$$**$$	−27,658.34$$***$$	−23,171.26
*Pension wealth*			
Gap(%)	15.9%	16.0%	18.9%
Male	26,157.32$$***$$	62,572.56$$***$$	196,332.49$$***$$
Female	21,991.29$$***$$	52,556.08$$***$$	159,214.21$$***$$
Difference	4166.03$$**$$	10,016.48$$***$$	37,118.28$$***$$
Explained	8,325.13$$***$$	23,592.94$$***$$	45,219.42$$***$$
Unexplained	−4,159.09	−13,576.45$$***$$	−8101.14
Observations	8138		

Estimated using the SOEPv30 2012 and 2013 sample including non-retired individuals ages between 25 and 60 and sample weights and excluding self-employed. Sample includes 3600 males and 4538 females. Percentages in terms of males. Decomposition estimated using the full set of controls described in Appendix A

$$*$$ $$p<0.05$$, $$**$$ $$p<0.01$$, $$***$$ $$p<0.001$$

**Table 23:** RIF-OAXACA decomposition of gender gap, population 25-60 including inheritance values

	(1)	(2)	(3)
	Q25	Q50	Q90
**Net Wealth**			
Gap(%)	38.6%	36.8%	27.2%
Male	6232.7$$***$$	48,755.2$$***$$	281,551.6$$***$$
Female	3,826.6$$***$$	30,832.6$$***$$	205,089.1$$***$$
Difference	2406.1	17,922.6$$***$$	76,462.5$$***$$
Explained	12,698.3$$***$$	34,567.1$$***$$	120,017.7$$***$$
Unexplained	−10,292.2$$**$$	−16,644.5$$**$$	−43,555.2$$*$$
Explained			
Inheritance	32.7	201.5	3277.8$*$
Unexplained			
Inheritance	−377.1$$***$$	−659.7$$**$$	−3,404.9
*Augmented wealth*			
Gap(%)	14.0%	20.3%	28.4%
Male	49,714.3$$***$$	129,923.7$$***$$	470,177.9$$***$$
Female	42,746.1$$***$$	103,533.9$$***$$	336,292.0$$***$$
Difference	6968.2$$*$$	26,389.8$$***$$	133,885.9$$***$$
Explained	33,897.8$$***$$	50,863.1$$***$$	150,136.3$$***$$
Unexplained	−26,929.7$$***$$	−24,473.3$$**$$	−16,250.4
Explained			
Inheritance	95.8	283.8	4444.7$*$
Unexplained			
Inheritance	−595.2$$***$$	−1256.8$$***$$	−1830.1
*Pension wealth*			
Gap(%)	8.3%	11.4%	17.9%
Male	24,387.4$$***$$	61,029.5$$***$$	196,981.7$$***$$
Female	22,351.5$$***$$	54,083.8$$***$$	161,756.0$$***$$
Difference	2035.9	6945.7$$**$$	35,225.7$$***$$
Explained	8987.0$$***$$	18,343.3$$***$$	41,800.2$$**$$
Unexplained	−6951.1$$**$$	−11,397.6$$**$$	−6,574.6
Explained			
Inheritance	−15.1	−8.6	31.6
Unexplained			
Inheritance	75.9	−108.7	−151.0
Observations	8894		

Estimated using the SOEPv30 2012 and 2013 sample including non-retired individuals between 25 and 60 years old. Each column includes the estimates for indicated wealth variable as the dependent variable. Decomposition estimated using the full set of controls described in Appendix A

$$*$$ $$p<0.05$$, $$**$$ $$p<0.01$$, $$***$$ $$p<0.001$$

## Footnotes

1 Private pension plans play a relatively small role in the German pension system and are a standard component of net worth. Public pension wealth has not been considered in previous research on the gender wealth gap.

2 However, there was also a wage gap in the GDR, which in 1989 amounted to 24% among full-time employees (Nickel, 1995).

3 Horizontal segregation exists when, for example, a particular industry is composed mainly of one gender, whereas vertical segregation exists when employees are not given a position above a certain threshold because of their gender.

4 This can be seen, for example, in the fact that single women achieve a higher retirement income in Germany and Britain than married women (Fasang et al., 2013).

5 The retirement age has been raised gradually from 65 to 67. The phase-in started with individuals born in 1947 and has been increased by one month per birth cohort and reached 67 for individuals born after 1963.

6 The details of these different pension plans are discussed in detail in Bönke et al. (2019).

7 Note that the survey does not ask explicitly about assets in checking accounts or cash, but these may be included in financial assets. Credit card debt—although relevant, for example, in the USA—does not play a major role in Germany.

8 This makes a total of 16,385 observations. Excluding 271 early pensioners and individuals younger than 25 and older than 60 leaves 8,894 observations, including 1,135 not employed or jobless.

9 The derivation of pension assets in the German case is quite straightforward, as the amount of the entitlements depends primarily on the number of pension points earned over the life course and not, as in other pension systems, for example, on earnings in the last years of employment or, in universal pension systems, on the number of years spent in a country.

10 We follow the analyses by Bönke et al. (2019) and Wolff (2015) by using a discount rate of 3%. The robustness of the result to using different discount rates is found in the former.

11 The derivation of occupational pensions can be challenging: This is especially true for defined benefit pensions which rely on the final earnings benefit formula (Luchak & Gunderson, 2000).

12 We refrain from decomposing the gap at the 10th percentile as net worth is zero at this point in the distribution.

13 Table 13 in Appendix shows that men are substantially more likely than women to be employed in manufacturing and construction, while women are more likely to be employed in wholesale and retail trade and health and social work. The distribution across company size is relatively even between men and women.

14 This is a standard regression based decomposition method used in the literature. More recently, Evelyn (Kitagawa, 1955) is also cited in relation to this literature. She developed a non-regression based decomposition method for rates acknowledged widely in the demographic and sociological literature.

15 The labour force participation rate of women in the 30–35 age group, for example, increased by 11 percentage points between 2005 and 2019, more than double the rate for men in the same age group.

16 See also Westermeier et al. (2017) on the trend of a declining gender pension gap by cohorts.

17 As an additional robustness check, we also present an alternative specification in Table 23 that includes inheritances. The results do not change significantly in terms of the size of the gap. The net wealth and augmented wealth gap increase slightly at the median in absolute terms. Inheritances are significant in explaining the gap at the top of the distribution favouring men. Yet the returns to inheritances are in favour of women at the 25th and 50th percentiles.

18 Since 2013, new marginal jobs below 450 euros per month are generally subject to compulsory insurance in the statutory pension scheme, but marginal workers can also opt out. In addition, it is now possible to make special payments to compensate for pension reductions starting at the age of 50. Prior to 2017, such payments were only possible starting at the age of 55. However, both reforms are unlikely to have any significant impact on the level of pension entitlements overall.

19 This data gap has now been filled by the SOEP group with a new sample of the very wealthy that was launched in 2019. Data on pension entitlements are not collected in this sample, however.
